# The interplay between prior selection, mild intermittent exposure, and acute severe exposure in phenotypic and transcriptional response to hypoxia

**DOI:** 10.1002/ece3.9319

**Published:** 2022-10-09

**Authors:** Millicent N. Ekwudo, Morad C. Malek, Cora E. Anderson, Lev Y. Yampolsky

**Affiliations:** ^1^ Department of Biological Sciences East Tennessee State University Johnson City Tennessee USA; ^2^ Ann Romney Center for Neurologic Diseases, Brigham and Women's Hospital Harvard Medical School Boston Massachusetts USA; ^3^ Department of Biological Sciences University of Notre Dame Notre Dame Indiana USA

**Keywords:** Daphnia, differential gene expression, hypoxia, lifespan, local adaptation, mitochondrial membrane potential, tolerance

## Abstract

Hypoxia has profound and diverse effects on aerobic organisms, disrupting oxidative phosphorylation and activating several protective pathways. Predictions have been made that exposure to mild intermittent hypoxia may be protective against more severe exposure and may extend lifespan. Here we report the lifespan effects of chronic, mild, intermittent hypoxia, and short‐term survival in acute severe hypoxia in four clones of *Daphnia magna* originating from either permanent or intermittent habitats. We test the hypothesis that acclimation to chronic mild intermittent hypoxia can extend lifespan through activation of antioxidant and stress‐tolerance pathways and increase survival in acute severe hypoxia through activation of oxygen transport and storage proteins and adjustment to carbohydrate metabolism. Unexpectedly, we show that chronic hypoxia extended the lifespan in the two clones originating from intermittent habitats but had the opposite effect in the two clones from permanent habitats, which also showed lower tolerance to acute hypoxia. Exposure to chronic hypoxia did not protect against acute hypoxia; to the contrary, *Daphnia* from the chronic hypoxia treatment had lower acute hypoxia tolerance than normoxic controls. Few transcripts changed their abundance in response to the chronic hypoxia treatment in any of the clones. After 12 h of acute hypoxia treatment, the transcriptional response was more pronounced, with numerous protein‐coding genes with functionality in oxygen transport, mitochondrial and respiratory metabolism, and gluconeogenesis, showing upregulation. While clones from intermittent habitats showed somewhat stronger differential expression in response to acute hypoxia than those from permanent habitats, contrary to predictions, there were no significant hypoxia‐by‐habitat of origin or chronic‐by‐acute treatment interactions. GO enrichment analysis revealed a possible hypoxia tolerance role by accelerating the molting cycle and regulating neuron survival through upregulation of cuticular proteins and neurotrophins, respectively.

## Graphical abstract

Mild intermittend hypoxia extends lifespan in Daphnia clones from intermittent ponds, but not from permanent ponds and does not provide added protection against acute severe hypoxia. Several pathways, including glycolysis and gluconeogenesis as well as cuticular proteins and neurotrophins are upregulated by acute hypoxia, in the manner consistent with hypoxia tolerance.

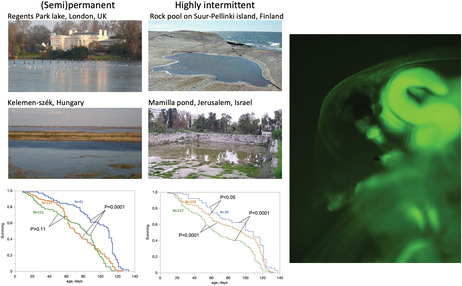

## 1 INTRODUCTION

A vast literature exists on local adaptation to hypoxia in terrestrial habitats where low oxygen partial pressure is a permanent feature of high altitudes (Brutsaert et al., 2019; Hochachka et al., 1998; Scott et al., 2015; Yi et al., 2010). Somewhat less is known about local adaptation and phenotypic plasticity in response to hypoxia in aquatic habitats, where oxygen availability is both lower and more variable than in the atmosphere. Dissolved oxygen (DO) concentration can vary from above the 8–10 mg/L saturation levels during peaks of photosynthetic activity to below 1 mg/L in warm, meromictic, and organic‐rich habitats (Díaz & Rosenberg, 2011; Gray et al., 2002). Hypoxic state of aquatic habitats is variably defined as dissolved O_2_ concentrations below 2 mg/L or below 30% of saturation (i.e., values at which acute mortality starts to be observed; Gray et al., 2002). Episodes of hypoxia occurring on both diel and seasonal scale are a major source of mortality in aquatic organisms and a cause of a significant economic impact (Díaz & Rosenberg, 2011).

In general, regulatory responses to hypoxia have been well characterized. Hypoxia‐mediated responses are controlled by a family of heterodimeric transcription factors, the hypoxia‐inducible factors (HIFs), which directly or indirectly activate numerous regulatory pathways and are conserved across Metazoa (Yeo, 2019). These pathways include SIRT1, FOXO, AMPK, mTOR, and NF‐κB pathways, all known to mediate stress response and affect longevity. (Antikainen et al., 2017; Gorr et al., 2010; Hong et al., 2014; Leiser et al., 2013; Pan & Finkel, 2017; Ruderman et al., 2010; Rui et al., 2011). There are several possible mechanisms through which these pathways can affect longevity; these include authophagy and protection against oxidative stress. Additionally, HIF‐1α exerts transcriptional control over genes associated with oxygen transport and homeostasis such as hemoglobins, erythropoietin, and vascular endothelial growth factor (*VEGF*), which are vital for increasing tissue perfusion and oxygenation as an adaptive response to hypoxia (Yeo, 2019; Zeis et al., 2009). HIF‐1α also upregulates lactate dehydrogenase (Firth et al., 1995) and, consequently, the conversion of pyruvate to lactate when low oxygen hinders membrane phosphorylation, thereby decreasing the NAD^+^/NADH ratio. Finally, hypoxia is known to induce inflammation and the immune response by increasing the expression of tumor necrosis factor‐alpha (TNF‐α; Scholz et al., 2013). A less understood role of a particular TNF, namely the p75 neurotrophin receptor protein (p75^NTR^) in hypoxia response, may include neurons survival during hypoxia‐ and reoxygenation‐caused oxidative stress (Sankorrakul et al., 2021).

As the data on the complex interplay between these diverse hypoxia‐initiated protective pathways has accumulated in the last two decades, it has become common to hypothesize that chronic exposure to mild and/or intermittent hypoxia may hormetically acclimate organisms to tolerating more severe or more prolonged hypoxic conditions (Hermes‐Lima et al., 2015; Visser et al., 2018) and also to extend lifespan (Blagosklonny, 2011; Yeo, 2019). The lifespan extension by mild hypoxia exposure of variable length has been shown in *C. elegans* (Leiser et al., 2013; Mehta et al., 2009), *Drosophila* (Harrison & Rascon, 2008), and mice (Kulikov et al., 2019), among others; importantly the life‐extending effect of hypoxia is dependent on the HiF‐regulation and can be reversed in mutants with overexpressed antioxidant response (Leiser et al., 2013). It is worth noting that senescence‐reducing effect of hypoxia is observed even when hypoxic conditions are experienced only early in life in *C. elegans* (Mehta et al., 2009) and in rats (Martin et al., 2012).

Species greatly differ in their ability to survive in hypoxic conditions; aquatic organisms are no exception. A vast literature exists on hypoxia response in fish; in particular we have learned a great deal about adaptation to hypoxia from studies of hypoxia tolerant species (Braz‐Mota & Almeida‐Val, 2021; Mandic et al., 2018; Sloman et al., 2008; Somo et al., 2020; Zhou et al., 2020) and from comparison of transcriptional responses between hypoxia‐tolerant species and their more hypoxia‐sensitive relatives (Heinrichs‐Caldas & De Almeida‐Val, 2021; Lau et al., 2019; Mandic et al., 2014). Coherently with the data from terrestrial vertebrates reviewed above, studies reported decreased oxygen consumption, metabolic depression and, transcriptionally, upregulation of glycolysis and amino acid degradation pathways, oxidative‐stress‐related enzymes and pathways, such as mitogen‐activated protein kinase (MAPK) signaling pathway, and apoptotis‐related genes, among others. Importantly, similar level of hypoxia tolerance can be achieved by different transcription‐level plastic responses (Mandic et al., 2018).

On the other hand, data on intraspecific heritable or phenotypic variation in hypoxia tolerance and hypoxia‐related transcriptional responses are fragmentary and largely limited to fish (Andersen et al., 2020; Anttila et al., 2013; Borowiec & Scott, 2020; Brennan et al., 2018; Crispo et al., 2020; Gorr et al., 2010; Regan et al., 2017; Shuang et al., 2022) and select invertebrates (Falfushynska et al., 2020; Gorr et al., 2010; Sandoval‐Castillo et al., 2018). Some of these studies report data consistent with rapid appearance of local adaptation to geographically variable hypoxia likelihood (Brennan et al., 2018; Pédron et al., 2017; Regan et al., 2017; Schulte, 2014). Data on the acclimation effects of mild or intermittent hypoxia that allow higher tolerance to more severe hypoxic treatments have indicated that at least some such hormesis effects exist (Borowiec & Scott, 2020; Hermes‐Lima et al., 2015; Peruzza et al., 2021; Yang et al., 2013). However, details of transcriptional and biochemical plasticity behind these acclimation effects are not well understood. Likewise, is not clear whether such responses show significant variation in nature. If such variation is present and consistent with hypoxia exposure in nature, this may reflect local adaptation, i.e., selective differentiation among populations based on environmental differences.

Even less is known about transcriptional responses to hypoxia in aquatic organisms. Data of this type have just started to accumulate, through both transcriptome‐wide studies (Feng et al., 2021; Flight et al., 2011; Hu et al., 2021; Jie et al., 2021; Kim et al., 2021; Mu et al., 2020; Tian et al., 2020; Xu, Fu, et al., 2021; Xu, Miao, et al., 2021; Zhou et al., 2020) and targeted qPCR‐based studies focused on anaerobic metabolism‐related genes (Aksakal & Ekinci, 2021; Amorim et al., 2021; Cota‐Ruiz et al., 2015; Reyes‐Ramos et al., 2018). These studies reveal that signals of some of the hypoxia‐inducible pathways described above are detectable in fish and invertebrates and that several downstream pathways showing response include anaerobic glycolysis, gluconeogenesis, and immune responses. Because these studies seldom analyze differential expression in different genotypes or geographically distinct populations (but see Flight et al., 2011; Kim et al., 2021; Zhou et al., 2020), the question of genetic variation for hypoxia response remains open.

The goal of this study was to address the question about the effecs of chronic exposure to mild hypoxia on lifespan and other fitness‐related phenotypes and on survival in acute hypoxia, in genotypes of different ecological backgrounds, using a model freshwater zooplankton organism, *Daphnia magna* Straus*. Daphnia* are particularly suitable for the studies of genotype‐by‐environment interactions because its cyclic parthenogenesis reproduction mode allows to expose identical genotypes to different environmental conditions and to eliminate genetic heterogeneity in lifespan measurement cohorts, at the same time allowing to study outbred genotypes rather than inbred lines. The availability of genomic data has made this classic physiological ecology model organism also an emerging model of choice for ecological genomics (Miner et al., 2012).

Short‐term phenotypic responses of *Daphnia* to hypoxia have been well characterized. Not surprisingly, the most obvious response is the upregulation of hemoglobin expression (Kimura et al., 1999; Kobayashi et al., 1990; Zeis et al., 2003, 2013; Zeis, 2020). Induction of hemoglobin expression occurs throught highly conserved the HIF‐1α mechanism (Colbourne et al., 2011; Gerke et al., 2011; Gorr et al., 2004; Klumpen et al., 2017; Pirow et al., 2001; Zeis et al., 2003), with the regulatory role of HIF‐1α expanding into broader metabolic responses, such as response to starvation (Klumpen et al., 2021). Depending on the level of hemoglobin expression, *Daphnia* are capable to maintain tissue‐ and lymph oxygen levels close to saturation down to 3–4 kPa O_2_ partial pressure (1–1.5 mg/L DO at room temperature and standard atmosphere; Baümer et al., 2002). Below this concentration, oxygen availability and appendage movement and heart beat rates quickly decline (Baümer et al., 2002; Pirow & Buchen, 2004). We will hereafter refer to this level of hypoxia as acute; it typically results in death in less than 24 h.

Concentrations even slightly above the 1.5 mg/L DO allow acclimation to hypoxic conditions resulting in long‐term survival (Seidl et al., 2005) with few changes in metabolism (Garreta‐Lara et al., 2018), physiology or life history (Andrewartha & Burggren, 2012). DO concentrations of 2–4 mg/L are experienced by Daphnia daily in their natural environments during vertical migrations to lower, more hypoxic water levels (Zeis et al., 2009). Rearing *Daphnia* at these DO concentrations results in moderate increase in mortality rate and decrease in body size and fecundity (Lyu et al., 2015; Seidl et al., 2005); these effects, as well as transcriptional responses (see below), may be trans‐generational (Andrewartha & Burggren, 2012; Lai et al., 2016) with maternal hypoxia exposure affecting offspring phenotypes. Importantly, a brief exposure to non‐lethal hypoxia may increase heat tolerance (Coggins et al., 2017), possibly through activation of antioxidant pathways (Becker et al., 2011; Klumpen et al., 2017). Since in nature hypoxia is often Iing intermittently during daily vertical migration, intermittent, rather than permanent exposure may be the most conducive to any beneficial effects on gene expression and physiology.

Previously published data on proteomics and transcriptional responses to chronic hypoxia in *Daphnia* embryos indicated upregulation of hemoglobin genes, HSPs, glutathione metabolism‐related transcripts, and downregulation of vitellogenins, histones, and histone‐modification‐related transcripts (Lai et al., 2016; Zeis et al., 2009), as well as glycolytic and other carbohydrates catabolism enzymes (Zeis et al., 2009). In a comparison between naturally occurring “red” (high hemoglobin) and “pale” (low hemoglobin), *D. pulicaria* that presumably represent, respectively, hypoxia‐ and normoxia‐exposed subpopulations, Lee et al. (2022) revealed few transcriptional changes, with the exception of, again, hemoglobins and lactate dehydrogenase. It is therefore entirely possible that mild and/or intermittent (i.e., sustainable) levels of hypoxia do not elicit major transcriptional changes in *Daphnia*, with the exception of a handful of genes with well‐characterized roles in hypoxia response. What transcriptional effect severe (i.e., lethal within 24 h) hypoxia may have in *Daphnia* is not known. Even less is known about potentially revealing interactions between exposures to mild and severe hypoxia, for example, whether acclimation to mild hypoxia may physiologically prepare *Daphnia* for higher tolerance of lethally hypoxic conditions.

Phenotypes we aimed to characterize in *Daphnia* acclimated to mild intermittent hypoxia included, besides long‐term survival, respiration and feeding rates, tissue lactate and pyruvate concentrations and mitochondrial membrane potential (as a measure of respiration intensity), expecting the increase in lactate/pyruvate ratio and decrease in mitochondrial membrane potential. We then tested the following hypotheses. (1) Acclimation to chronic mild intermittent hypoxia (CMIH thereafter) can extend lifespan through activation of antioxidant and stress‐tolerance pathways. (2) Acclimation to CMIH can increase survival in the condition on acute severe hypoxia (thereafter ASH). (3) Transcriptional response to CMIH can set stage for more radical transcriptional changes in response to ASH, i.e., we expect CMIHxASH interactions in transcriptional profile. 4. Transcriptional responses, besides well‐expected hemoglobin and lactate metabolism expression changes, will include genes involved in stress‐response pathways.

We conducted the experiments using four geographically distinct *D. magna* clones originating from natural habitats that differ in their hydrological and hydrochemical characteristics in order to capture at least some intraspecific variability in prior history of selection. All four water bodies sampled are shallow water bodies, typical for *D.magna* natural habitats, but differed greatly in their size (ranging from <10 m^2^ to up to 4.8 km^2^) and salinity. An important characteristic of the two smaller habitats (a rock pool in Finland and a Mediterranean pond in Israel that differed them from the other two, much larger water bodies, was that these two habitats predictably dry every summer). Both rock pools (Ganning, 1971; Jocque et al., 2010) and Mediterranean intermittent ponds (Zacharias et al., 2007) are known for significant daily and seasonal oscillations of DO concentration, particularly during summer dry season. It is reasonable to assume that *Daphnia* originating from these habitats have been under a stronger selection favoring hypoxia adaptation than those originating from more permanent water bodies. Previous studies (Anderson et al., 2022; Coggins et al., 2021) indicated that the two clones from the intermittent habitats had lower lifespan and higher propensity for sexual reproduction than the two clones from more permanent water bodies, so we anticipated differences in lifespan effects of hypoxia.

Indeed, post hoc, it was discovered that many of the physiological and transcriptional effects differ between the clones from intermittent, hypoxia‐prone habitats, and those originating from more permanent, hypoxia‐free habitats, thus indicating local adaptation. However, many of these differences, apart from lifespan, were not among our a priori expectations and thus not based on planned comparisons; additionally, we only characterized two clones per habitat type. Thus, any results reported here that pertain to local adaptation to habitat type must be taken with caution.

## 2 MATERIALS AND METHODS

### 2.1 Clones' provenance and maintenance

Details on the geographic origins of the four clones used in this study are listed in Table 1. Clones were obtained from Basel University (Switzerland) Daphnia Stock Collection (D. Ebert, personal communication) and propagated asexually in the lab in COMBO water (Kilham et al., 1998) at 20°C under a 16:8 D:L light cycle and fed by a diet of *Scenedesmus acutus*. Prior to all experiments, experimental cohorts were established through the following procedure: Five randomly selected grandmother females from each clone were maintained from birth till their third clutch of offspring were born at the density of one individual per 20 ml of COMBO water with *Scenedesmus* food added daily to the concentration of 10^5^ cells/ml and water changed every 4 days. Offspring from their second and third clutches were used to establish the maternal generation, which was in turn maintained in the same conditions until enough offspring from the second or consecutive clutches could be collected to form the experimental cohorts. Neonates were maintained in groups of 20 in 200 ml jars with COMBO water for the first 6 days of their lives until transferred to the corresponding experimental tanks.

**TABLE 1:** Location of origin of *Daphnia* clones used. First two letters used in the text as clone ID

Clone identifier	Location	Latitude	Longitude	Habitat type	Surface area	Depth	Salinity/pH	Dries up every summer
FI‐FSP1‐16‐2	Rock pool on Suur‐Pellinki island, Finland	60° 10′ 04″	25° 47′ 41″	Intermittent summer rock pool	<10 m^2^	<0.3 m	Low/Low	Yes
IL‐M1‐8	Mamilla pond, Jerusalem, Israel	31° 46′ 40″	35° 13′ 14″	Intermittent Mediterranean summer‐dry pond	<2500 m^2^	<0.5 m	Low/Low	Yes
GB‐EL75‐69	Regents Park lake, London, UK	51° 31′ 40″	−0° 9′ 35″	Permanent lake^a^	Main basin 0.3 km^2^	1.2 m	Low/Low	No^b^
HU‐K‐6	Kelemen‐szék Fülöpszállás, Hungary	46° 47′ 33″	19° 10′ 54″	Semi‐permanent soda lake^a^	Varies, up to 4.8 km^2^	<0.4 m	High/high	No^c^

^a^ Both water bodies are variably described as “lakes” or “ponds”.

^b^ Last completely drained in 1867.

^c^ Dries up periodically, but not every summer.

### 2.2 Chronic mild intermittent hypoxia treatment

**FIGURE 1:**
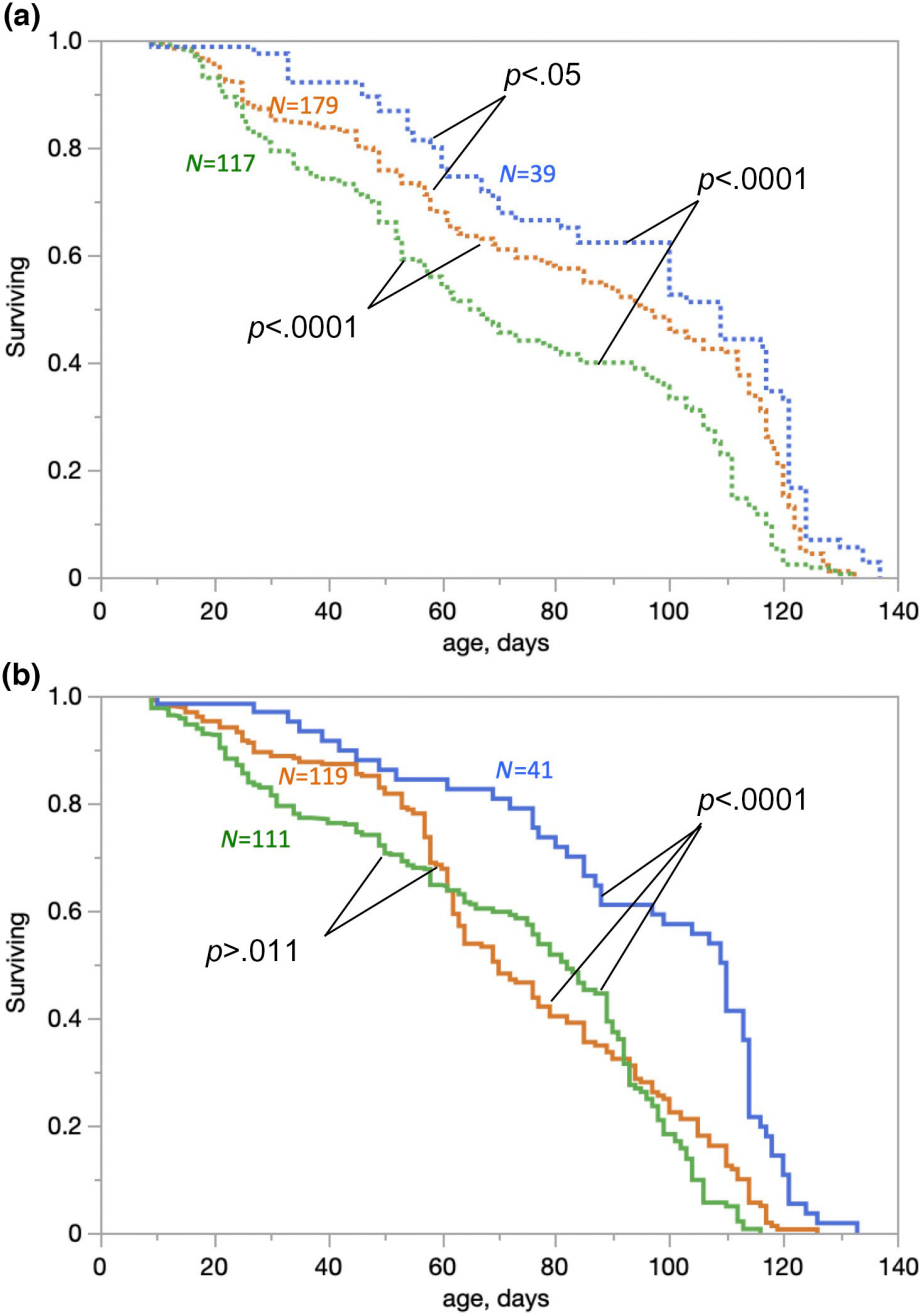
Survival curves of *Daphnia* from intermittent habitats (a, dashed lines) and permanent habitats (b, solid lines) in normoxic conditions (8 mgO_2_/L, green), chronic mild intermittent hypoxia (4 mg O_2_ /L twice daily; orange), or after switch from 4 to 8 mgO_2_/L at day 30 (blue). Clones within habitat types were not a significant factor in the proportional hazards analysis and are polled together here, as are replicate tanks within each treatment. P values for Log‐rank test for survival differences between groups and cohort sizes (N) are shown. See Table 2 for detailed survival analysis. See Figure S4 for the same data grouped by hypoxia conditions rather than by habitats of origin.

*Daphnia* cohorts were maintained in 5‐L tanks each containing eight plastic containers with 1 mm nylon mesh bottoms, which allowed free water exchange and the removal of neonates during water changes. Cohort sizes are shown on Figure 1. Water volume and daily food ratios were adjusted every 4 days to maintain 20 ml of water per individual, and 10^5^ *Scenedesmus* cells were added per mL per day. The water was changed, neonates removed, and a census of *Daphnia* cohorts conducted every 3 days. Chronic mild intermittent hypoxia treatment (CMIH) was achieved by bubbling N_2_ through the experimental tanks with continuous monitoring of oxygen concentration by the Extech DO210 probe (Nashua, NH, USA) twice daily, at lights on and at lights out times, until the concentration was lowered to 4 mg/L. This concentration was chosen as the CMIH treatment as the concentration likely to be experienced by *Daphnia* on a daily basis in natural habitats, close to ~50% of saturation and twice the concentration in which acute mortality had been observed in preliminary experiments. Twice daily adjustment to 4 mg/L O_2_ roughly simulated natural exposure to hypoxia that typically occurs either at night when photosynthesis stops, while plant respiration continues, or during the day when *Daphnia* migrate to lower, less oxygenated layers of water. At the same times, the control tanks were aerated with ambient air until the oxygen concentration reached 8 mg/L. Between these procedures, the oxygen concentration in the hypoxia tanks typically raised to 6.5 mg/L by diffusion, whereas the control tanks typically experienced a drop in oxygen concentration of 7–7.5 mg/L due to respiration of *Daphnia* or its aerobic metabiome (Figures S1–S4). At the age of 100 days (at about 20% survival), replicate tanks were combined in order to maintain constant volume per remaining individuals without dropping water level close to the containers' mesh bottoms. Due to space and handling time limitations, the CMIH experiment was conducted in two blocks, with each block consisting of two CMIH and two control tanks.

To investigate whether early life exposure to mild hypoxia provides the same protection as lifetime exposure, an additional replicate tank was set up within the CMIH treatment, which was switched to normoxic condition on day 30 of the experiment. In the absence of prior data on which might constitute early life exposure, the switching point was chosen to occur before any significant mortality in the cohort occurred. Because, due to space and handling time constraints, this switch treatment included only one replicate tank, the results of this comparison will be interpreted as highly speculative and preliminary.

At various time points, subsets of individuals from each cohort were sampled (with replacement) for the measurements of body size (*N* = 158), fecundity (*N* = 62), feeding rate (*N* = 39), and respiration rate (*N* = 120) measurements or for the measurements of lactate and pyruvate concentration (*N* = 112), acute hypoxia tolerance experiment (*N* = 204), or RNAseq (without replacement). See below for more detailed samples sizes for each clone in each treatment. Additionally, hemoglobin concentration in tissues was measured in *Daphnia* from CMIH and normoxic control treatments by means of light microscopy (with replacement) and (without replacement) by light absorption in homogenates (Ekwudo, 2021). These measurement showed the expected increase of hemoglobin concentration in the CMIH treatment (Ekwudo, 2021) and are not reported here.

### 2.3 Lactate and pyruvate measurements

Lactate and pyruvate assays were conducted using CellBiolab fluorometric kits (catalog #s MET‐5013 and MET‐5029) in 15–20‐day‐old and 55‐60‐day‐old *Daphnia* from two clones (GB and IL) sampled from the experimental tanks and stored frozen at −80°C until assay time, sampling 25–32 replicates per clone per CMIH treatments spread over two blocks (96‐well plates). Each *Daphnia* was homogenized in 100 μl ice‐cold PBS with a pestle, and the homogenates were centrifuged at 4°C. 25 μl of supernatant was pipetted into each of the lactate and pyruvate assay plates using the manufacturer's protocols. Additionally, two replicate aliquots of 15 μl of the supernatant each were used to quantify soluble proteins by Bradford assay, with 185 μl of Bradford colorimetric reagent added to each well. All assay well plates were analyzed using a BIOTEK plate reader (Agilent). Lactate and pyruvate fluorescence assay sensitivity was set to 35, and the Bradford assay absorption was measured at 595 nm.

### 2.4 Respiration and filtering rates and fecundity measurements

Respiration rate was measured in individual *Daphnia* sampled from the cohorts, 12 replicates per clone per treatment for the FI and HU clones and 18 replicates per clone per treatment for the IL and GB clones, due to availability. As described in Anderson et al. (2022), *Daphnia* were individually placed in either 200 μl or 1700 μl 24‐well respirometry glass plates (Loligo®), after which the plates were sealed with PCR sealing tape, and oxygen concentration measured using SDR fluorescence sensors (PreSens). Either normoxic (8 mg O_2_/L) or hypoxic (4 mg O_2_/L) COMBO water was used in these measurements. Each measurement continued for 45–60 min, with oxygen concentration recorded every 15 s until the oxygen concentration dropped by at least 1 mg/L. The first 15 min after the transfer of *Daphnia* into the respirometry plates were discarded as the break‐in period. The slope of the linear regression of oxygen concentration over time was used as the measure of oxygen consumption rate.

Feeding (filtering) rate was measured by placing individual *Daphnia* (five replicates per clone per CMIH treatment; four in one combination of clone and treatment where one individual was killed during handling) into plastic 24‐well plates with normoxic COMBO water containing the initial concentration of 2 × 10^5^ *Scenedesmus* cells per mL and measuring chlorophyll fluorescence at the start and after 8, 12, and 24 h of filtering activity.

Fecundity was measured by sampling four individuals from each of the mesh bottom cups in each of the CMIH experiment tanks, i.e., 32 individuals per clone per treatment. The four individuals from the same container were considered technical replicates, and their clutch sizes were averaged, thus resulting in eight independent estimates per clone per treatment. After egg counting, *Daphnia* were immediately returned into their experimental containers.

After both respiration and feeding rate measurements, *Daphnia* body lengths were measured to the precision of 0.035 mm and returned to the containers. Body length was used to estimate body weight using previously obtained regression (Coggins et al., 2017; Figure S2) to be used as a normalization factor for respiration rates and lactate and pyruvate concentrations.

### 2.5 Mitochondrial membrane potential

Samples for mitochondrial membrane potential (ΔΨ_m_) measurements were taken from the cohorts at the median lifespan of all cohorts, i.e., at the age of 80 days to maximize longevity‐related effects. Sixteen individuals were sampled per clone per CMIH treatment; in two cases some replicates were lost, resulting in 12 and 8 replicates. ΔΨ_m_ was measured by means of rhodamine‐123 assays as described in Anderson et al. (2022). Briefly, *Daphnia* were exposed for 24 h to a 4 μM solution of rhodamine‐123 dye, washed three times, and photographed using a Leica DM3000 microscope with a 10× objective (0.22 aperture) equipped with a Leica DFc450C color camera, with a 488 nm excitation/broadband (>515 nm) emission filter. The fluorescence from the following tissues and organs was measured: antenna‐driving muscle, epipodite, brain, and optical lobe, with the median intensity of fluorescence used as the measure of ΔΨ_m_. We have previously shown that ΔΨ_m_ does not change much with age in most tissues (Anderson et al., 2022), so it is likely that these measurements are representative of any age of *Daphnia* exposed to CMIH for a sufficient time.

### 2.6 Acute severe hypoxia experiments and sample collection for RNA‐Seq

For acute severe hypoxia (ASH) tolerance measurement, 25‐days‐old *Daphnia* were sampled from each of the four tanks in one of the two CMIH experiment blocks. This sampling occurred 3 h after the CMIH O_2_ concentration has been adjusted (to 4 mg/L in CMIH and to 8 mg/L in normoxia). Six individuals per clone per CMIH treatment were immediately frozen in liquid nitrogen, forming the no acute exposure controls. Thirty‐five individuals per clone per treatment were moved into 70 ml cell culture flasks filled with COMBO water with the concentration of oxygen at or below 1 mg/L, sealed without air bubbles, seven *Daphnia* per flask, five replicate flasks per clone per treatment. The DO concentration of 1 mg/L was chosen as the ASH treatment as a measurable concentration below the acute hypoxia cutoff of 1.5 mg/L that seems to be critical in *Daphnia* (Baümer et al., 2002; Pirow & Buchen, 2004). Flasks were kept at 20°C and checked for mortality 12 h after the onset of the experiment and every hour thereafter.

Simultaneously, six individuals per clone per treatment were set aside in the same flasks but with either 8 or 4 mg/L O_2_ concentration without food to serve as RNAseq controls not exposed to acute hypoxia treatments. These controls and two randomly chosen individuals sampled from the ASH flasks frozen in liquid nitrogen after 12 h of exposure (before any mortality occurred in the ASH treatment). After sampling the ASH treatment flasks were then topped with 1 mg O_2_/L water and sealed again. Survival time analysis was performed by proportional hazards model with CMIH treatment and habitats as main effects with clones nested within habitats (all effects fixes) using the Survival platform of JMP statistical package (Ver. 16; SAS Institute, 2021), and by the same model with CMIH and habitats as fixed effects, clones as random effect nested within habitats and flasks as a random effect nested within clones using coxme R package (Therneau et al., 2003). Individuals sampled for RNAseq were censored from the cohort.

### 2.7 RNA sequencing

RNA was extracted and cDNAs sequenced from *Daphnia* from the four combination of CMIH and ASH treatments for each of the four clones, with three replicates per clone per treatment combination, total 48 libraries. The four treatments were in full factorial fashion with respect to CMIH and ASH: *Daphnia* reared at normoxia; *Daphnia* reared at normoxia and exposed to acute hypoxia for 12 h; *Daphnia* reared in CMIH; and *Daphnia* reared in CMIH and exposed to acute hypoxia for 12 h. *Daphnia* for RNAseq were taken directly from the CMIH and ASH experiments described above (with appropriate censoring of survival data); each replicate consisted of two individuals. RNA was extracted using Qiagen RNAeasy kit (Cat ID: 74134) and quantified using a Qubit (ThermoFisher) fluorometer.

Following extraction, RNAs were reverse transcribed and sequencing libraries were constructed from the cDNAs as prescribed by the Oxford Nanopore Technology (Oxford, UK) PCR‐cDNA Barcoding kit protocol (SQK‐PCB109). Barcoded samples from the four treatments within each clone were pooled together into three replicate libraries, purified separately, and pooled together immediately before adding the sequencing adapter. Libraries were then sequenced using Oxford Nanopore MinION for 24–48 h per sequencing run, obtaining 2–4 Gb of reads in each run.

### 2.8 RNAseq data analysis

Basecalling and reads filtering, demultiplexing, trimming, and mapping were accomplished using ONT Guppy software (ver. 3.6). *Daphnia magna* reference transcriptome 3.0 (D.Ebert and P.Fields, NCBI BioProject ID: PRJNA624896) was used as a reference. For the purpose of this analysis, the reference transcriptome containing only the longest isoform for each gene was used, with a total of 33,957 transcripts, of which at least one read mapped to 22,445 transcripts. The distribution of read length and the numbers of reads per sample are shown on Figure S3. Transcripts were then filtered to retain only those that contained at least 72 reads across all samples, resulting in a set of 6050 transcripts retained for further analysis. This number was chosen to allow retaining of 6 K transcripts or more, a compromise between reducing false negatives by including as many transcripts as possible without over‐inflating the totals for multiple test correction purposes. DESeq2 (see below) does not require pre‐filtering of low read features other than for more efficient memory use and for visualization purposes (Love et al., 2014; http://bioconductor.org/packages/devel/bioc/vignettes/DESeq2/inst/doc/DESeq2.html).

As each library and each sequencing run consisted of three biological replicates of each of four combinations of CMIH and ASH treatments from a single clone, clones were fully confounded with replicate library preparation and sequencing runs. The advantage of this design is that each library preparation contains a balanced set of all four treatments with a clone‐library replicate combination that can be used as a block effect in the Likelihood Ratio tests (see below). The disadvantage of this design is the lack of the ability to test for the difference among clones, since it does not allow untangling the variance among clones from random variance among library preparation and sequencing runs.

For the purpose of visualization, read counts normalized as RPKMs (reads per kilobase of reference sequence per million reads) were used in principal component analysis (PCA; SAS Institute, 2021). Only transcripts with at least one potentially significant effect in the LRT model were used in this analysis; there were 587 such transcripts.

Differential expression was analyzed using Likelihood Ratio tests (LRT) in DESeq2 (Love et al., 2014) with CMIH treatment, ASH treatment, and habitat of origin as orthogonal effects and clones as an effect nested within habitats. In DESeq2 analysis, log‐fold change noise was shrunk by the *apeglm* algorithm for the Wald test (Zhu et al., 2019). DESeq2 analysis was conducted separately for full data, for the two habitats of origin separately, and for the analysis of the CMIH factor for the subset of samples not exposed to ASH treatment (ASH = Control). For LRT analysis, the reduced model consisted of all factors except the factor being tested and all its interactions. Wald tests and LRTs yielded similar results; with only LRT results reported. An arbitrary adjusted *p*‐value of *p*_adj_ < .05 was chosen as the cutoff for reporting differential gene expression in any given transcript. R scripts are available in the Supplementary materials.

Enrichment analysis was conducted separately for lists of candidate transcripts with a possible significant effect of each of the main effects (CMYH, ASH, habitat type) and of their interactions, using an arbitrary cutoff of an uncorrected *p* < .01. We used two approaches to generating reference annotation data. Firstly, we constructed a non‐overlapping list of transcripts with annotation terms that included pathways and functions a priori known to play a role in hypoxia response. The only deviations from the non‐overlapping principle were the fused genes containing vitellogenin and superoxide dismutase domains (Kato et al., 2004), which appeared both in the “vitellogenins” and “antioxidant pathway” gene lists. The source of annotation was a combination of descriptions obtained from the *D. magna* genome annotation available at (http://arthropods.eugenes.org/genepage/daphniamagna/Dapma7bEVm000001t1; Gilbert, 2002) and annotations obtained for the *D. magna* 3.0 transcriptome by blast2GO (Götz et al., 2008) and PANTHER (Mi et al., 2013) software. The advantage of this approach to annotation was the ability to include a priori known genes implicated in pathways a priori expected to be under hypoxia control. The disadvantage of this approach was the inability to discover pathways not identified from literature and reliance on unreliable genomic annotations.

Secondly, we used all GO annotations generated by blast2GO and PANTHER to construct a reference list of all GOs; this list was filtered to include only gOs represented in the reference transcriptome by at least 10 transcripts. These lists were overlapping, i.e., the same transcript could (and typically did) occur on more than one list. The advantage of this approach was that it included all GOs to which *Daphnia* transcripts map with some degree of certainly. The disadvantage of this approach was high expectation of false positives due to mismatching of transcripts to GO based on sequence similarity alone.

In both cases, the left‐handed Fisher exact test was used to test for enrichment, testing the hypothesis that a given pathway, function, or GO had a higher than random representation in the list of candidate transcripts.

## 3 RESULTS

### 3.1 Lifespan in chronic mild intermittent hypoxia

CMIH treatment provided a life‐extending effect in clones originating from intermittent habitats but not in clones originating from permanent habitats (Figure 1; Table 2). The median lifespan increased from 65 days (60–73, 95% CI) to 97 days (85–106, 95% CI) in clones from intermittent habitats. In the clones from permanent habitats, the median lifespan decreased slightly and not statistically significantly from 82 days (76–89 95%CI) to 70 days (64–77 95%CI). The proportional hazards test of the habitat × hypoxia interaction term had a marginally significant *p* < .032 and only when clones were treated as fixed effects; it was not significant in the coxme analysis with clones as a random nested effect (Table 2); likewise, the clone x hypoxia interaction was not significant (Table 2). Analyzed for each habitat type separately, the CMIH treatment effect was significant for the clones from intermittent habitats, but not from permanent habitats, with some evidence of CMIHxClone interactions (Table 2).

**TABLE 2:** Likelihood ratio proportional hazards tests of the effects of habitat of origin type, clones nested within habitats, hypoxic conditions, and their interactions on *Daphnia* lifespan (Figure 1)

Lifespan in CMIH vs. normoxic control; clones treated as a nested fixed effect			
Source	DF	Log ratio *χ* ^2^	*p* value
Habitat	1	57.29	<.0001
Clone[habitat]	2	4.46	.11
CMIH	1	10.57	.0011
Habitat*CMIH	1	4.58	.032
Clone*CMIH [habitat]	2	3.01	.22

Lifespan in CMIH vs. normoxic control; clones treated as a nested random effect (coxme)			
Source		*z*	*p* value
Habitat		3.39	.0007
CMIH		−2.11	.035
Habitat*CMIH		0.28	.78

Lifespan in CMIH vs. normoxic control vs. CMIH‐ > normoxia switch			
Source	DF	Log ratio *χ* ^2^	*p* value
Habitat	1	40.87	<.0001
Clone[habitat]	2	2.35	.31
CMIH	2	51.12	<.0001
Habitat*CMIH	2	6.34	.042
Clone*CMIH [habitat]	4	8.34	.080
Habitat of origin: intermittent			
Clone	1	2.44	.12
CMIH	2	12.14	.0005
Clone*CMIH	2	8.272	.016
Habitat of origin: permanent			
Clone	1	2.76	.10
CMIH	2	1.30	.25
Clone*CMIH	2	4.47	.035

*Note*: Top: comparison of CMIH (4 mg O_2_/L twice daily) to normoxic (8 mg O_2_/L) control with clones treated as either fixed or random nested effect. Middle: same with the switch from CMIH to a normoxic regime at day 30 included. Bottom: the same analysis was conducted separately for each habitat type. In the analysis with the switch treatment included, the significant effect of hypoxia treatment is due to both the difference between the switch and CMIH treatment (*p* < .05) and between the switch and control (*p* < .0001).

The switch from CMIH to normoxic treatment provided further extension of lifespan in all clones (Table 2), increasing median lifespan to 109 days (100–117 95%CI) in clones from intermittent habitats and to 110 days (88–113 95%CI) in clones from permanent ones. Switch vs. constant treatments contrasts in proportional hazard tests with “switch” as one of the levels of hypoxia factor revealed that the effect of the switch was significant in clones from intermittent habitats in the switch vs. normoxia contrast (model parameter = 0.384 ± 0.0694) but not in the switch vs. CMIH treatment contrast (model parameter = −0.0214 ± 0.066). In clones from permanent habitats, both contrasts were significant (0.261 ± 0.071 and 0.359 ± 0.074 in switch to CMIH and switch to control contrasts, respectively).

### 3.2 Body size, fecundity, feeding rate, and respiration rate in CMIH vs. control

**FIGURE 2:**
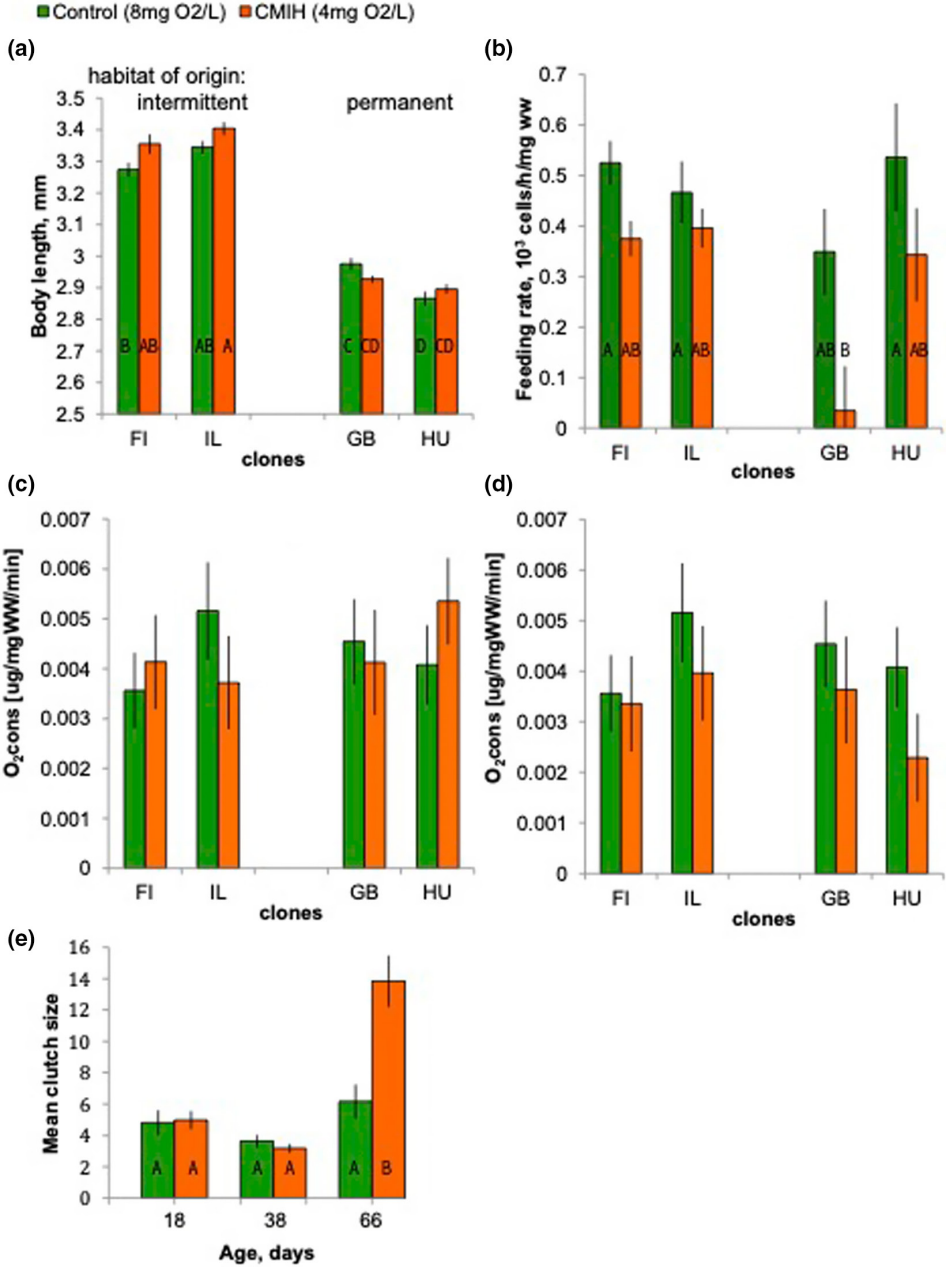
Body length at age 18 days (a), feeding rate (b), respiration rate (c, d) and fecundity (e) in four *Daphnia* from intermittent (FI, IL) and permanent (GB, HU) habitats reared either in normoxic conditions (8 mgO_2_/L, green) or chronic mild intermittent hypoxia (CMIH, 4 mg O_2_/L twice daily; orange). Respiration rate is measured either at an initial O_2_ concentration of 8 mg/L (c) or at an initial O_2_ concentration equal to that in the rearing conditions (d). The bars here and below are standard errors. Letters on bars indicate significant differences among groups in Tukey test, *p* < .05: groups sharing a letter are not different. Not shown when no differences are significant. See Table 1 for clones' provenance and Table 3 for full statistics.

Chronic mild intermittent hypoxia had a small and marginally significant effect on *Daphnia* body size, but there was a significant cloneXhypoxia interaction effect (Figure 2a, Table 3): both clones from permanent habitats (which had lower body size in controls) became even smaller in hypoxic conditions, while those from intermittent habitats became larger. *Daphnia* reared in CMIH had a lower feeding (filtering) rate than their normoxic control counterparts (Figure 2b, Table 3). This result held regarding whether the feeding rate was normalized by the wet weight of *Daphnia* or not. In particular, CMIH‐reared *Daphnia* from the GB clone had an almost undetectably low feeding rate in this experimental setup. In contrast, respiration rate did not differ between CMIH and control *Daphnia* (Figure 2c,d, Table 3), either with an oxygen assay conducted with an initial oxygen concentration of 8 mg/L (Figure 2c) or with *Daphnia* from each CMIH treatment tested with the initial oxygen concentration matching their rearing conditions (Figure 2d).

**TABLE 3:** Two‐way ANOVA of the effects of hypoxia and clones (random effect) on body length at maturity, feeding rate, and respiration rate. Habitat types, with clones nested with habitats are pooled when not significantly different

Response: body length at age 18 days				
Source	DF	MS	*F* ratio	Prob > *F*
Hypoxia	1	0.0366	4.40	.038
Clone	3	2.4795	298.3	<.0001
Hypoxia*clone	3	0.0341	4.10	.008
Error	151	0.0080		
Response: feeding rate, wet weight normalized				
Hypoxia	1	0.331	12.24	.0014
Clone	3	0.155	5.73	.003
Hypoxia*clone	3	0.026	0.95	.43
Error	32	0.027		
Response: respiration rate				
AssayO2	1	22.90	2.047	.16
Hypoxia	1	0.12	0.011	.92
AssayO2*hypoxia	1	0.12	0.010	.92
Clone	3	2.72	0.243	.87
AssayO2*clone	3	0.84	0.075	.97
Hypoxia*clone	3	2.63	0.236	.87
AssayO2*hypoxia*clone	3	12.29	1.099	.35
Error	101	11.18		
Response: respiration rate, measured at acclimation O_2_ concentration				
Hypoxia	1	19.78	2.29	.13
Clone	3	7.21	0.84	.48
Hypoxia*clone	3	1.75	0.20	.89
Error	70	8.63		
Response: mean clutch size at age 18 days				
Hypoxia	1	0.22	0.0276	.87
Clone	3	11.20	1.3898	.27
Hypoxia*clone	3	2.00	0.2482	.86
Error	24	8.06		
Response: mean clutch size at age 38 days				
Hypoxia	1	0.84	1.86	.21
Clone	3	2.95	6.54	.015
Hypoxia*clone	3	0.62	1.37	.32
Error	8	0.45		
Response: mean clutch size at age 66 days				
Hypoxia	1	242.67	32.1	.0013
Clone	3	7.98	1.06	.43
Hypoxia*clone	3	27.61	3.65	.08
Error	6	7.56		

Finally, early fecundity did not differ between CMIH and control treatment, but at the age of 66 days, CMIH‐reared *Daphnia* showed significantly higher clutch sizes than those in the control (Figure 2e, Table 3).

### 3.3 Survival in severe acute hypoxia

**FIGURE 3:**
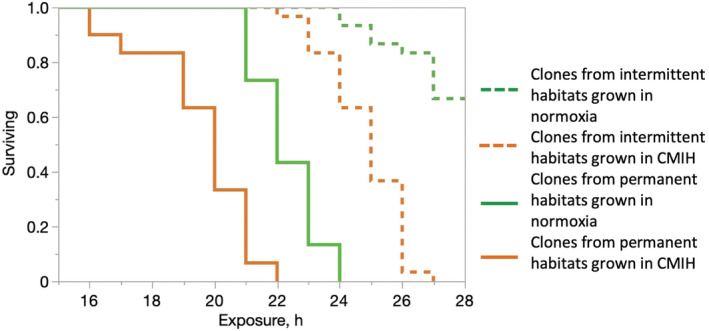
Survival in acute severe hypoxia (ASH; <1 mg/L O_2_) of *Daphnia* from intermittent habitats (dotted lines) and permanent habitats (solid lines) reared in normoxia (green) or in chronic mild intermittent hypoxia (CMIH; 4 mg O_2_ /L; orange). See Table 4 for detailed survival analysis.

Contrary to the predictions about protective or preconditioning acclimatory effects of CMIH on hypoxia tolerance, *Daphnia* from the CMIH treatment showed lower acute hypoxia tolerance in ASH trials than their normoxia‐reared counterparts (Figure 3, Table 4). Clones from intermittent habitats had a significantly higher ASH tolerance than those from permanent ones (Figure 3, Table 4), with clones within habitat types being only marginally significantly different from each other.

**TABLE 4:** Likelihood ratio proportional hazards tests of the effects of habitat type, chronic hypoxia treatment (CMIH), and their interactions on *Daphnia* acute hypoxia survival, with replicate flasks nested within clones within habitats as random nested effects (Figure 4).

Fixed effects	*Z*	*p*
Habitat	3.39	.00071
CMIH	−2.11	.035
Habitat*CMIH	0.28	.78
Random effects		
Group	Variance	
Clone/Flask	0.9409	
Clone	0.0567	

### 3.4 Lac/Pyr ratio

**FIGURE 4:**
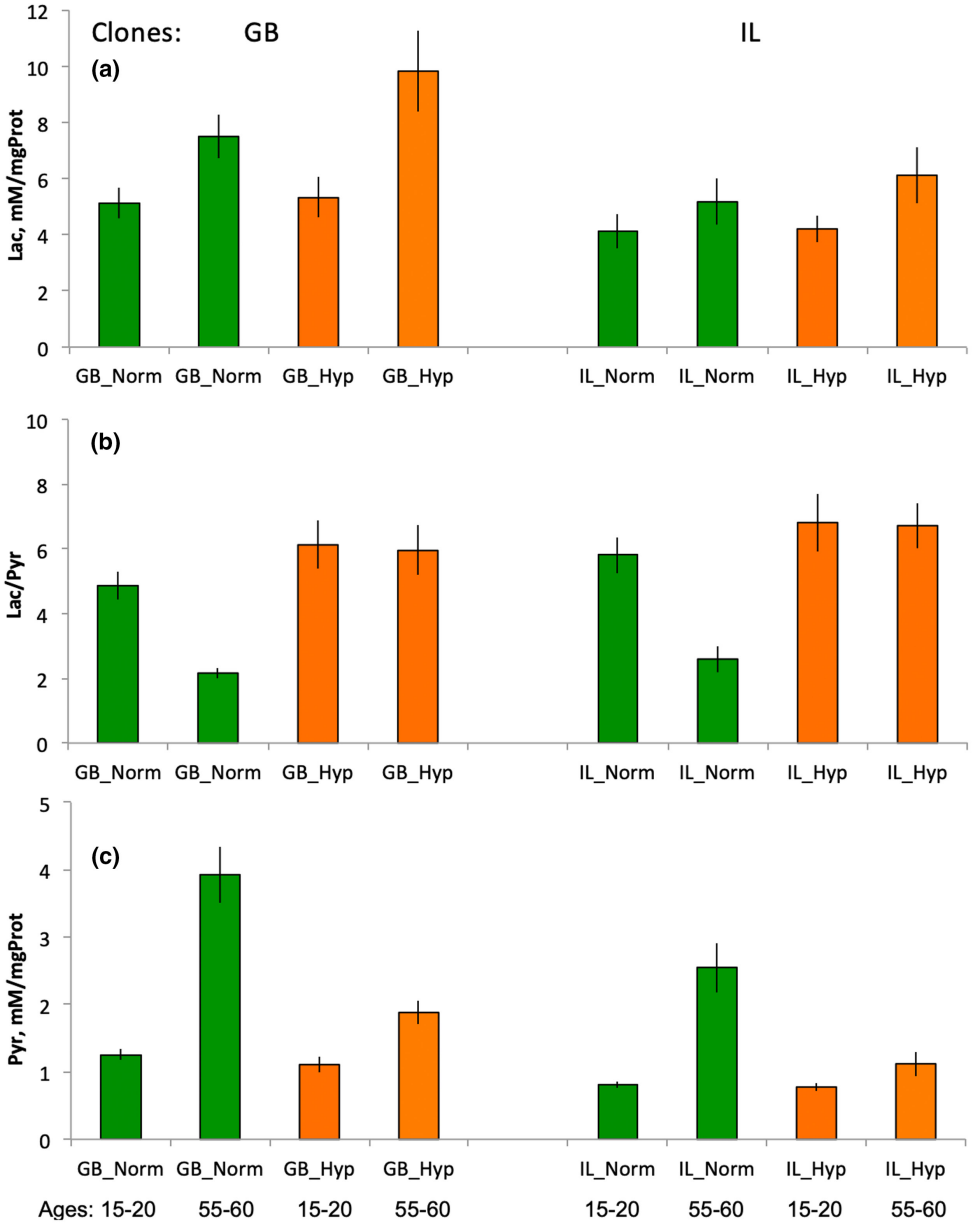
Whole body lactate/pyruvate ratio (a) and protein‐normalized lactate and pyruvate concentrations (b, c) in young (15–20 days) and moderately aged (55–60 days) *Daphnia* from the two clones studied reared at either normoxic control (green) or CMIH (orange) conditions. See Table 5 for statistics.

As expected, *Daphnia* reared in CMIH conditions showed an elevated Lac/Pyr ratio relative to normoxic controls (Figure 4, Table 5). This difference was especially noticeable in older (55–60‐day‐old) *Daphnia* (Figure 4a) and was primarily due to a decrease in pyruvate concentration (Figure 4c) rather than an increase in lactate concentration (Figure 4b). Contrary to the prediction of decreased NAD+ availability with age (i.e., an increase in lactate concentration with age), the Lac/Pyr ratio dropped significantly between age classes of 15–20 and 55–60 days. This drop, again, was largely explained by the increased pyruvate concentration (Figure 4c; Table 5). *Daphnia* reared in the CMIH condition showed no change in Lac/Pyr ratio with age, with a slight increase in both protein content‐normalized lactate and pyruvate concentrations with age, resulting in a significant (*p* < .0001) CMIHxAge interaction (Table 5).

**TABLE 5:** The effects of CMIH, age, and clones on the Lac/Pyr ratio (Figure 3). “Plate” is a random block effect. Habitat type not tested because data for only one clone per habitat type are available.

Source	DF	SS	*F* ratio	Prob > *F*
CMIH	1	252.8	51.31	<.0001
Clone	1	21.6	4.38	.0389
CMIH*clone	1	1.26	0.26	.61
Age	1	137.4	27.89	<.0001
CMIH*age	1	112	22.75	<.0001
Clone*age	1	0.21	0.04	.84
CMIH*clone*age	1	0.06	0.01	.92
Plate	1	107.8	21.88	<.0001
Error	103	507.79		

Response Pyr, mM/mgProt				
Source	DF	MS	*F* ratio	Prob > *F*
CMIH	1	49.36	4.82	.03
Clone	1	90.05	8.80	.0037
CMIH*clone	1	8.99	0.88	.35
Age	1	0.022	0.002	.96
CMIH*age	1	40.45	3.95	.049
Clone*age	1	15.90	1.55	.21
CMIH*clone*age	1	7.49	0.73	.39
Plate	1	109.65	10.72	.0014
Error	103	10.23		

Response Lac, mM/mgProt				
Source	DF	MS	*F* ratio	Prob > *F*
CMIH	1	23.51	20.97	<.0001
Clone	1	15.0	13.38	.0004
CMIH*clone	1	1.08	0.96	.33
Age	1	28.23	25.18	<.0001
CMIH*age	1	19.17	17.10	<.0001
Clone*age	1	3.44	3.072	.083
CMIH*clone*age	1	0.56	0.50	.48
Plate	1	0.85	0.75	.38
Error	103	1.12		

### 3.5 Mitochondrial membrane potential

**FIGURE 5:**
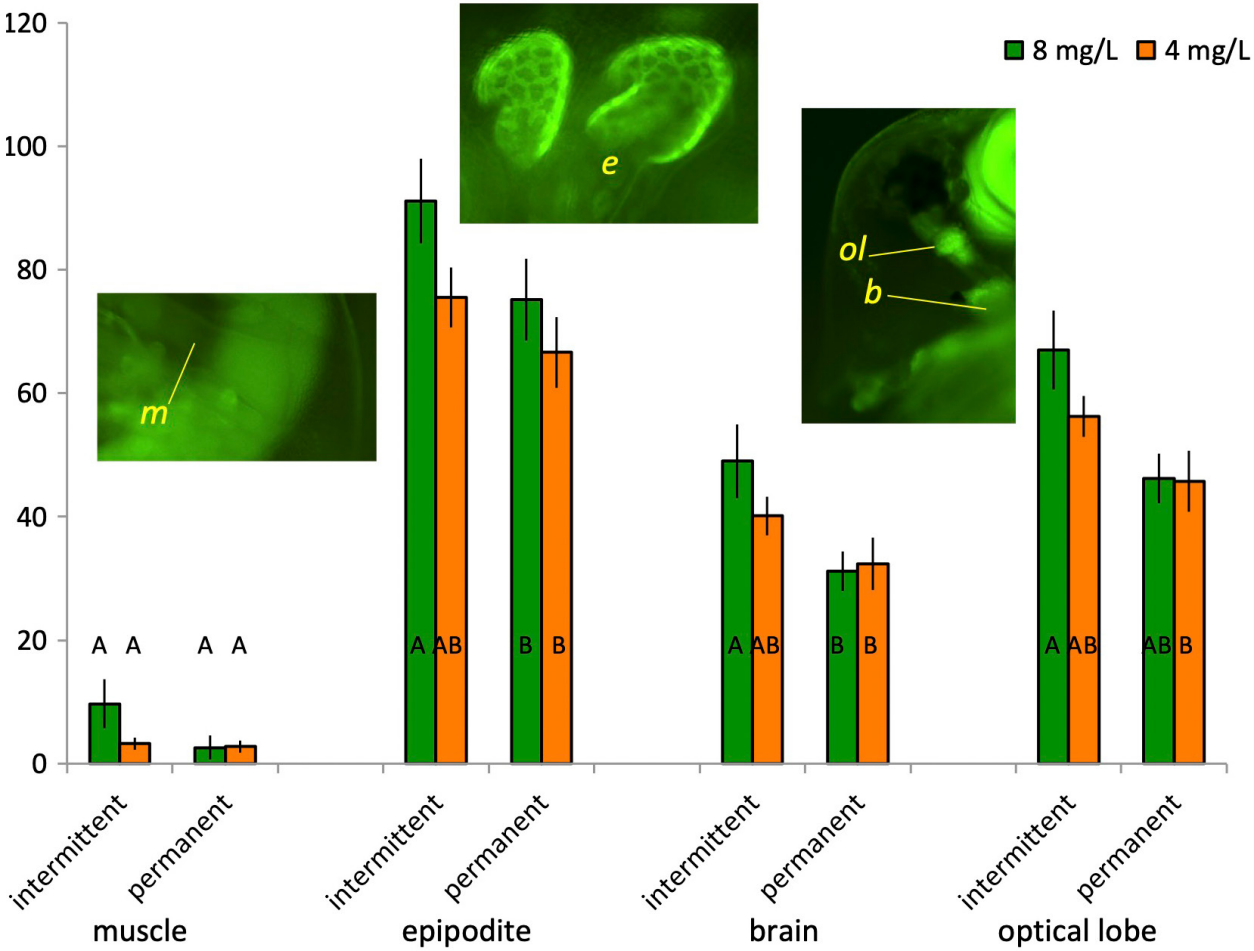
Median rhodamin‐123 fluorescence used as a measure of mitochondrial membrane potential in antenna‐driving muscle (m), epipodite (osmoregulation/gas exchange organ, e), brain (b), and optical lobe (ol) in *Daphnia* from either intermittent or permanent habitats reared either in normoxic (8 mg O_2_/L, green) or CMIH (4 mg O_2_/L twice daily, orange) conditions. Letters on the bars are the results of Tukey test conducted for each tissue separately. See Table 6 for statistical analysis.

ΔΨ_m_ was slightly higher in clones from intermittent habitats than in clones from permanent habitats across all four studied tissues (Figure 5, Table 6). Furthermore, clones from intermittent habitats tended to reduce their ΔΨ_m_ in the CMIH treatment more strongly than clones from permanent habitats, although individual pairwise comparisons were not significant in post‐hoc Tukey test. As a result, there was a significant difference between clones from the two habitat types in control, but not in CMIH treatment, at least in the epipodite and the brain. There was therefore no evidence that hypoxia‐tolerant genotypes showed any ΔΨ_m_ patterns specifically in CMIH conditions that would be different from those of hypoxia‐sensitive ones or correlate with the extended lifespan.

**TABLE 6:** Differences in mitochondrial membrane potential (ΔΨ_m_) among tissues, between CMIH treatments (CMIH vs. control), between habitat of origin (intermittent vs. permanent), and between clones nested within habitat type (Figure 5).

Source	DF	SS	*F* ratio	Prob > *F*
Tissue	3	73617.2	195.5	<.0001
CMIH	1	1129.7	9.00	.0035
Habitat	1	3773.9	30.06	<.0001
CMIH*Habitat	1	670.3	5.34	.0232
CMIH*Tissue	3	294.0	0.78	.508
Habitat*Tissue	3	590.6	1.57	.203
CMIH*Habitat*Tissue	3	29.0	0.077	.9722
Clone[Habitat]	2	1142.3	4.55	.0132
Clone*Tissue[Habitat]	6	462.1	0.61	.7189
Clone*CMIH[Habitat]	2	1659.6	6.61	.0021
Error	86	10796.1		

*Note*: Tukey test for differences among clones (*p* < .05): all pairwise comparisons of clones across habitat types significant, except IL vs. HU (*p* > .25).

### 3.6 Differential gene expression in response to CMIH and ASH: multidimentional analysis

**FIGURE 6:**
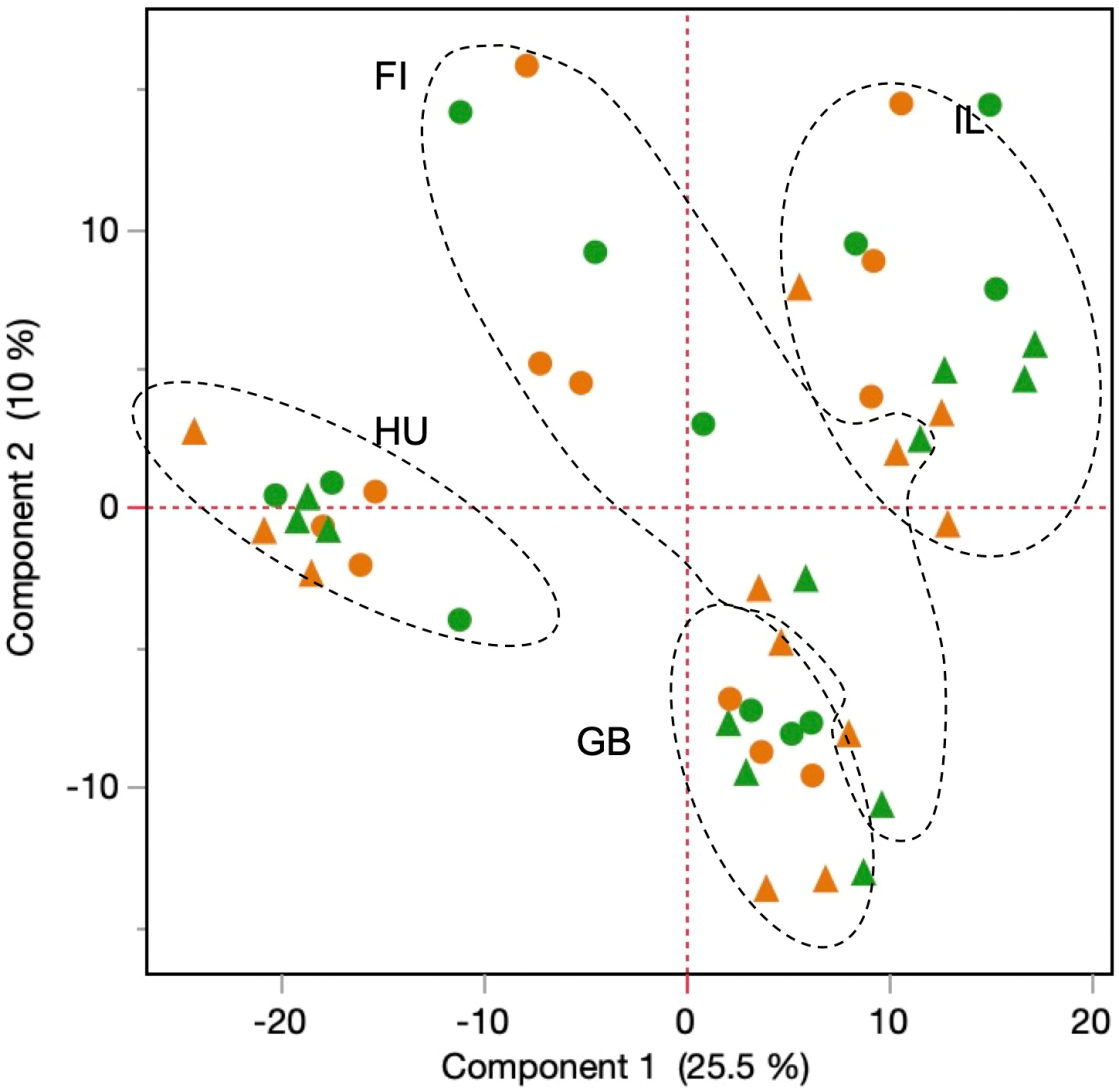
PCA of 48 samples (2CMIH × 2ASH × 4clones/libraries × three biological replicates) in the space of 587 transcripts with at least one uncorrected *p* < .01. Colors as on previous figures, namely green: CMIH control, orange: CMIH treatment. Circles: ASH control, triangles: ASH treatment. Ellipses encircling clone/library blocks drawn by hand. Clones from intermittent habitats: FI, IL; from permanent habitats: GB, HU. See Table 1 for clones' provenance and Table 7 for statistics.

Principal component analysis of 48 samples (2CMIH × 2ASH × 4clones/libraries × three biological replicates) in the space of 587 transcripts with at least one uncorrected *p* < .01 is shown in Figure 6. Several patterns are apparent from this analysis. Firstly, there were strong differences among confounded clone/library replicates. Secondly, within each such replicate, there was a variable degree of separation between ASH and control biological replicates (Figure 6, triangles vs. circles), but not between CMIH vs. normoxic control (orange vs. green), consistent with fewer transcripts reaching significant differential expression (see below). Thirdly, this separation was significantly stronger in the clones from intermittent habitats (FI and IL) than in those from permanent habitats (GB and HU): all three first principal components showed a significant separation of ASH vs. control in FI and IL, controlling for random differences between clones/libraries, but only the third principal component showed such separation in the GB and HU clones analyzed separately (Table 7).

**TABLE 7:** Mixed model two‐way ANOVA of the coordinates in the first three principal components in the space of 578 transcripts with at least one significant non‐adjusted *p*‐value. CMIH and ASH as fixed effects, clones as a random block. Analysis for clones from: intermittent and permanent habitats of origin separately. *p*‐values less than .001 shown in bold.

Habitat of origin				Intermittent		Permanent	
	Source	df	df_Den_	*F* ratio	Prob > *F*	*F* ratio	Prob > *F*
Response: PC1	CMIH	1	19	2.3416	.14	0.4789	.50
	ASH	1	19	16.226	**.0007**	1.877	.18
	CMIH*ASH	1	19	0.0562	.82	0.0677	.80
Response: PC2	CMIH	1	19	0.1161	.74	0.0519	.82
	ASH	1	19	18.6566	**.0004**	0.7377	.40
	CMIH*ASH	1	19	0.0091	.93	0.0015	.97
Response: PC3	CMIH	1	19	6.6618	.0183	3.4484	.079
	ASH	1	19	40.3107	**<.0001**	34.7833	**<.0001**
	CMIH*ASH	1	19	0.0036	.95	0.0031	.96

Finally, while the hypoxia‐tolerant clones from intermittent habitats (FI and IL) were somewhat separated from the hypoxia‐sensitive ones from permanent habitats (GB and HU), the direction of change from control to ASH treatment did coincide with this difference. Exposed to acute hypoxia, the sensitive clones did not change expression patterns to match the tolerant clones; rather, the hypoxia‐tolerant clones exposed to acute hypoxia became more transcriptionally similar to the sensitive ones (.). In other words, most of the differential expression did not reflect adaptive plasticity and/or canalization of transcriptional phenotype in the hypoxia‐tolerant clones.

### 3.7 Differential gene expression in response to CMIH and ASH: individual transcripts

**FIGURE 7:**
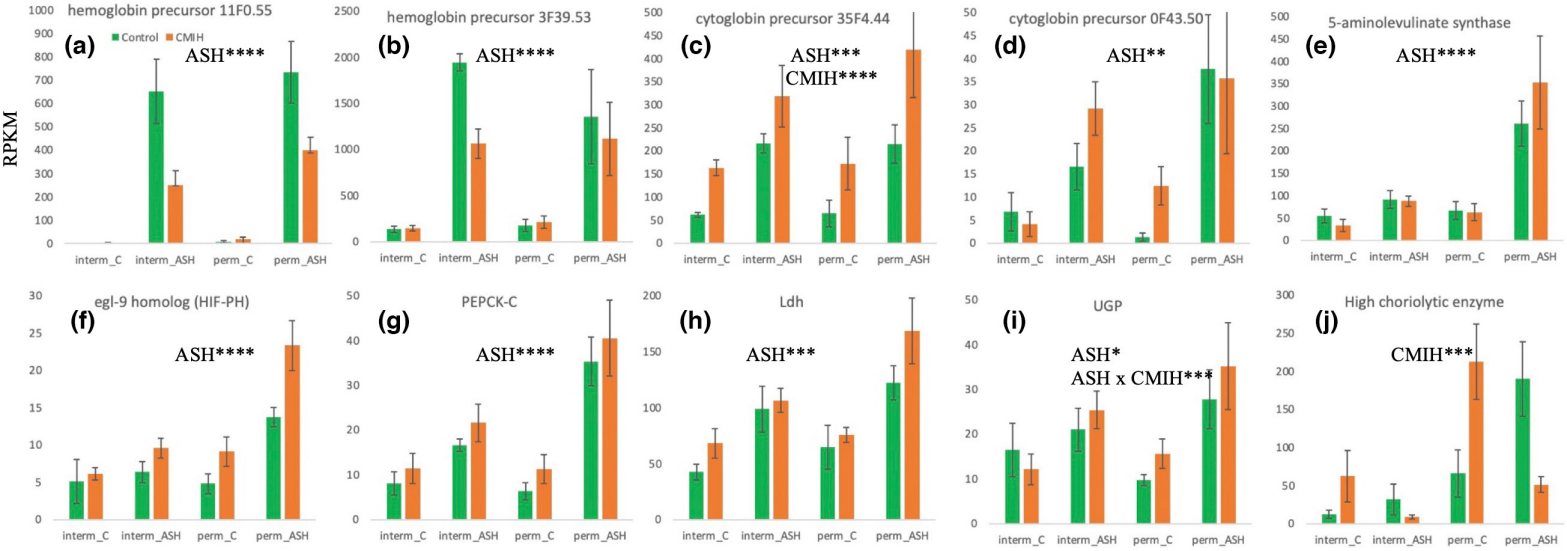
Read abundance (RPKM) in select transcripts with a significant (*p*_adj_ < .1) effect of CMIH or ASH in the 3‐way LRT analysis. *: *p*_adj_ < .1; **: *p*_adj_ < .01; ***: *p*_adj_ < .001; ****: *p*_adj_ < .0001. (a–e) hemoglobins, cytoglobins, and heme synthesis‐related genes. (f) HiF prolyl hydrolase. (g–i) pyruvate metabolism and gluconeogenesis‐related genes. (j) choriolytic enzyme.

Only three transcripts individually survived multiple test correction in the three‐way analysis of responses to CMIH (Table 8), all three showing upregulation in the CMIH treatment. This result did not change when the CMIH effect was analyzed within the ASH = Control subset (i.e., in *Daphnia* never exposed to acute hypoxia only) or in the analyses with each habitat of origin type separately (data not shown). These transcripts were (Table 8, Figure 7): one of the cytoglobin paralogs (a homolog of mammalian CYGB globin known to protect against hypoxia, probably by facilitating diffusion of oxygen through tissues and scavenging nitric oxide or reactive oxygen species; Trent & Hargrove, 2002) and two proteases, a chymotrypsin BI‐like protease and a high choriolytic enzyme zinc metalloprotease (with no known hypoxia‐related functions). Additionally, the latter transcript was the only one showing a significant CMIHxASH interaction (Table 8, Figure 7).

**TABLE 8:** Transcripts with a significant differential expression in response to either acute or chronic intermittent hypoxia (and their interaction), in all four clones and in clones from intermittent and permanent habitats separately. Direction column indicate up‐ or downregulation in the hypoxic treatment.

Whole dataset results	3‐way analysis		Separately in clones from each habitat type: Intermittent permanent	
Transcript description	*p*_adj	direction	*p*_adj	*p*_adj
Response to chronic hypoxia (CMIH)				
Cytoglobin	2.6E‐08	UP	4.0E‐05	
Chymotrypsin BI‐like	1.5E‐04	UP	0.051	
High choriolytic enzyme, zink metalloprotease	6.1E‐04	UP		0.002
Response to acute hypoxia (ASH)				
Di‐domain hemoglobin precursor	5.7E‐50	UP	2.8E‐29	8.7E‐22
DC‐STAMP domain containing protein	8.2E‐25	UP	3.0E‐12	8.5E‐12
Growth and transformation‐dependent protein	3.2E‐13	UP	1.2E‐08	2.0E‐06
Pleckstrin homology domain containing protein	2.5E‐11	UP	6.1E‐10	2.9E‐08
Di‐domain hemoglobin precursor	2.1E‐10	UP	6.6E‐11	2.8E‐16
Phosphoenolpyruvate carboxykinase, cytosolic	6.0E‐08	UP	N.S.	4.7E‐08
Low‐density lipoprotein receptor ldl	1.0E‐07	UP	N.S.	1.1E‐05
Zygote‐specific class v copy b gene protein	1.4E‐07	UP	N.S.	6.0E‐05
5‐aminolevulinate synthase, mitochondrial	3.3E‐07	UP	N.S.	1.4E‐08
Presequence protease, mitochondrial	2.0E‐06	UP	N.S.	0.007
Calcium‐binding mitochondrial carrier protein Aralar1	6.0E‐06	UP	8.3E‐03	N.S.
CAP‐Gly domain‐containing linker protein	6.7E‐05	UP	N.S.	N.S.
egl‐9 homolog (HIF‐PH)	6.9E‐05	UP	N.S.	0.029
4‐aminobutyrate aminotransferase, mitochondrial	2.3E‐04	UP	N.S.	N.S.
Persulfide dioxygenase ETHE1, mitochondrial	2.7E‐04	UP	9.9E‐08	3.41E‐04
L‐lactate dehydrogenase A chain	2.7E‐04	UP	N.S.	N.S.
Sphingosine kinase	5.4E‐04	UP	N.S.	N.S.
Cytoglobin	0.001	UP	1.0E‐12	9.70E‐09
Cytoglobin	0.004	UP	N.S.	N.S.
Phosphatase 1 regulatory subunit 3E	0.007	UP	N.S.	N.S.
RWD domain‐containing protein	0.017	UP	2.1E‐03	N.S.
UTP‐glucose‐1‐phosphate uridylyltransferase	0.023	UP	N.S.	N.S.
Normal mucosa of esophagus‐specific gene 1 protein	0.037	UP	3.7E‐08	2.66E‐08
CMIHxASH interaction	*p*_adj			
High choriolytic enzyme, zink metalloprotease	.00029			

Significant in clones from intermittent habitats, but not in the 3‐way analysis	*p*_adj direction
Endoribonuclease‐like protein	6.2E‐09 UP
Endoribonuclease‐like protein	4.0E‐08 UP
Alpha‐amylase	1.5E‐04 UP
Chitinase	0.011 UP
Ribonuclease t2	0.012 UP
Pleckstrin domain containing protein	0.026 UP
Alpha‐amylase	0.047 UP
Zinc carboxypeptidase a 1	0.048 UP
Sushi, von Willebrandd factor, egf and PTX domain‐containing protein	0.048 UP
Carboxypeptidase b‐like	0.048 UP
Asparagine synthetase domain‐containing protein	3.2E‐03 DOWN
Uncharacterized protein	8.1E‐03 DOWN
Brefeldin A‐inhibited guanine nucleotide‐exchange protein	0.027 DOWN

Separately in clones from permanent habitats, but not in the 3‐way analysis	*p*_adj direction
T‐cell activation inhibitor, mitochondrial	9.9E‐05 UP
High choriolytic enzyme	2.2E‐03 DOWN
Ubiquitin carboxyl‐terminal hydrolase	0.019 DOWN
Vitellogenin fused with superoxide dismutase	0.058 DOWN

*Note*: *p*adj < .05 used as the cutoff. N.S. = *p*_adj_ > .05, or in rare cases, the LRT model failed to converge.

A significantly longer, fully non‐overlapping list of transcripts showed a significant response to ASH treatment, both in three‐way LRT analysis and LRT analysis conducted separately in clones from the two habitat types (Table 8; Figure 7), again, all showing upregulation in ASH condition. Prominent in this list were the di‐domain hemoglobin precursors well characterized for their plastic response to hypoxia (Zeis, 2020; Zeis et al., 2009), as well as cytoglobins and mitochondrial 5‐aminolevulinate synthase critical for heme synthesis (Figure 7a,b,e), and not surprisingly, one of the homologs of egl‐9 (HIF prolyl hydroxylase) present in *Daphnia* (Figure 7f). Other transcripts upregulated in ASH include those coding for rate‐limiting enzymes in anaerobic metabolism such as lactate dehydrogenase (Ldh) and cytosolic phosphoenolpyruvate carboxykinase (PEPCK‐C; Figure 7g,h). There were also numerous ASH‐upregulated transcripts with mitochondrial membrane localization and functionality, as well as many with unclear relation to hypoxia and respiration, including several proteases. A different set of transcripts changed expression in the ASH treatment in the hypoxia‐tolerant, intermittent habitat clones analyzed separately (Table 8). Among them there were: calcium‐binding mitochondrial carrier protein Aralar1, several endoribonucleases, several α‐amylases, a chitinase, numerous carboxypeptidases, and one of several paralogs of SOD.

### 3.8 Differential gene expression in response to CMIH and ASH: Functional enrichment

Targeted analysis of differential expression within the set of a priori defined genes with hypoxia‐related functions revealed a significant enrichment of several a priori defined pathways or functions among transcripts with uncorrected *p*‐values in one or several types of LRT analysis (Table 9). Antioxidant pathways (represented mostly by SOD paralogs) as well as globins showed enrichment in LRT analysis of all factors in the model. Respiratory, electron transport chain, and ATP synthesis pathways did so in the analysis of the ASH effect and, with a less significant FDR value in the CMIHxASH interaction analysis. Other targeted functionalities were more specific to individual analyses. General hypoxia‐related (such as the egl‐9 paralog), MAPK‐related proteins and vitellogenins appeared to be specific to clones from permanent habitats. In contrast, HSPs, Lac/Pyr metabolism‐related proteins, as well as NFkB‐related and TNF‐related proteins were enriched in the analyses specific to clones from intermittent habitats. It appears that clones from different habitats engaged radically different repertoire of plastic transcriptional responses to either CMIH or ASH treatments. Notably, none of the other hypoxia‐related pathways showed any enrichment in any of the analyses, including FOXO‐, autophagy, AMPK, mTOR, and p53‐related pathways or the sirtuins (Table 9).

**TABLE 9:** Representation of a priori hypoxia‐related functions of interest in gene lists with uncorrected *p*‐values < .01 for CMIH, ASH, and their interactions in the whole data and estimated for clones originated separately from intermittent and permanent habitats

Function of interest	*N* in reference	ASH	CMIH	ASHxCMIH	ASH, habitat = intermittent	ASH, habitat = permanent	CMIH, habitat = intermittent	CMIH, habitat = permanent
Antioxidant pathways	33	**1.9E‐08**	**7.0E‐08**	**7.0E‐08**	**6.8E‐05**	**1.9E‐06**	**0.0090**	**0.0006**
Hemoglobins and cytoglobins	9	**5.9E‐08**	0.0756	0.0756	**1.2E‐08**	**1.9E‐06**	**2.0E‐05**	0.0746
Respiration/ETC/ATP synthesis	212	**8.9E‐06**	n.s.	0.0617	**0.0050**	**0.0037**	n.s.	n.s.
HiF‐related	5	**0.0023**	0.0756	n.s.	0.0618	**0.0014**	n.s.	0.0672
MAPK‐related	5	**0.0029**	n.s.	0.0567	n.s.	**0.0018**	n.s.	0.0896
Vitellogenins	12	**0.0082**	n.s.	n.s.	n.s.	**0.0039**	n.s.	**0.0001**
HSPs	25	0.0260	n.s.	n.s.	n.s.	n.s.	**0.0072**	n.s.
FOXO‐related	9	n.s.	n.s.	n.s.	n.s.	n.s.	n.s.	0.0639
Lac/Pyr metabolism	9	n.s.	n.s.	n.s.	0.0895	n.s.	n.s.	n.s.
NFkB‐related	7	n.s.	n.s.	n.s.	0.0959	n.s.	n.s.	n.s.
TNF‐related	5	n.s.	n.s.	0.0756	n.s.	n.s.	**0.0007**	n.s.
Autophagy‐related	25	n.s.	n.s.	n.s.	n.s.	n.s.	n.s.	n.s.
AMPK‐related	2	n.s.	n.s.	n.s.	n.s.	n.s.	n.s.	n.s.
Carbonic anhydrases	4	n.s.	n.s.	n.s.	n.s.	n.s.	n.s.	n.s.
mTOR‐related	16	n.s.	n.s.	n.s.	n.s.	n.s.	n.s.	n.s.
p53‐related	4	n.s.	n.s.	n.s.	n.s.	n.s.	n.s.	n.s.
PGC1a	4	n.s.	n.s.	n.s.	n.s.	n.s.	n.s.	n.s.
Sirtuins	3	n.s.	n.s.	n.s.	n.s.	n.s.	n.s.	n.s.

*Note*: Values are FDR‐adjusted Fisher exact test *p*‐values of significant overrepresentation (bold: FDR < 0.01).

**FIGURE 8:**
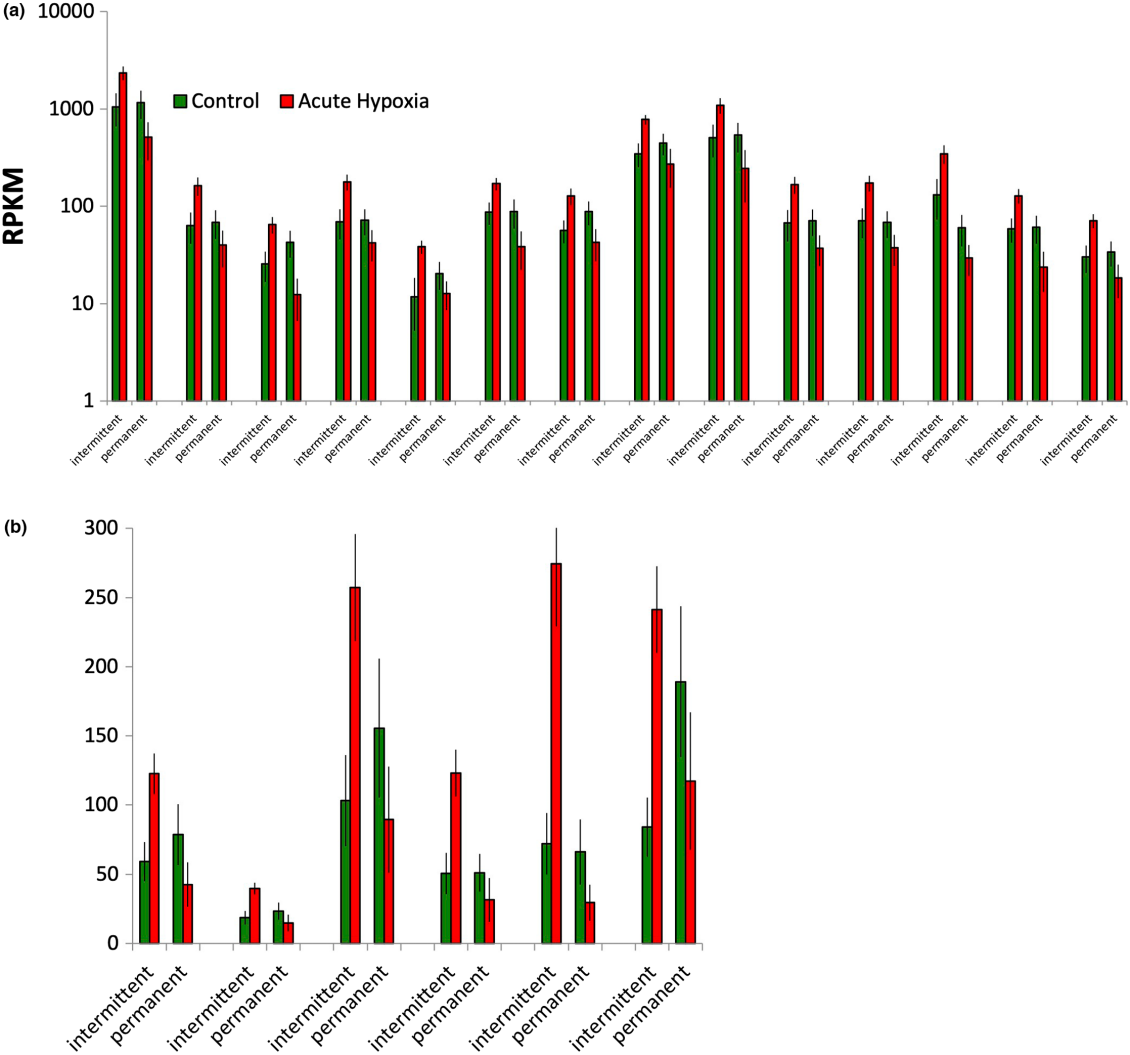
RPKM values of transcripts whose GOs are significantly enriched in the gene set with possible ASH × habitat type interactions. (a) 14 paralogs of cuticulum proteins; (b) six paralogs of neurotrophins. Both groups of transcripts show upregulation in *Daphnia* from intermittent habitats and downregulation in those from permanent habitats.

In the analysis of open GO list enrichment, few gene ontologies or pathways identified by blast2go and PANTHER scans showed any significant enrichment among any gene lists with possible differential expression (although many showed a significant under‐representation; results not shown). There were several possible exceptions overrepresented in a single gene set: those with an uncorrected *p*‐value <.01 for the ASHxhabitat type interaction. These GOs included various nested GO categories already revealed by the previous analysis, such as heat shock proteins or mitochondria‐related GOs. There were, however, also two types of GO or pathway categories not revealed by the previous analyses. Firstly, there was a significant enrichment for extracellular matrix structural proteins (PC:00103) PANTHER category and extracellular region cellular localization GO category (GO:0005576), both of which were overrepresented due to the high occurrence of cuticular proteins (Figure 8a). Secondly, central nervous system development category (GO:0007417) was overrepresented due to a group of paralogous neurotrophins (Figure 8b), encoded by a set of linked genes. Both these groups of transcripts show upregulation in *Daphnia* from intermittent habitats and downregulation in those from permanent habitats. None of the transcripts reached a significant FDR for the ASH x habitat interaction, but both GOs are overrepresented with FDR < 0.0001.

## 4 DISCUSSION

### 4.1 Chronic mild intermittent hypoxia extends lifespan in Daphnia from intermittent habitats

We observed an expansion of lifespan in CMIH treatment in clones from hypoxia‐prone, intermittent habitats, but not in clones from permanent habitats that rarely experience hypoxic conditions. While this can be readily interpreted as local adaptation, we did not see, consistently with Lee et al. (2022), any manifestation of it on the transcriptional level other than the upregulation of one of the cytoglobin paralogs in the clones from intermittent habitats, but not in those from permanent habitats. We must therefore conclude that, with the exception of the cytoglobin expression, this intriguing opposite directionality of CMIH on lifespan is not based on transcriptional profile differences among genotypes. Likewise, we did not observe any differences in respiratory phenotypes (respiration rate, lactate/pyruvate ratio, and mitochondrial membrane potential, ΔΨ_m_) interpretable as possible causes of lifespan extension in intermittent habitat clones in mild hypoxia. Respiration rate showed no significant differences at all; lactate/pyruvate ratio and ΔΨ_m_ showed differences between CMIH treatment and control consistent with reduced aerobic respiration in CMIH, but no differences between clones with and without lifespan extension effect. Higher ΔΨ_m_ in shorter‐lived clones from intermittent habitats apparent in the normoxic control is consistent, at least for the epipodite ΔΨ_m_, with the data reported in Anderson et al., 2022, as well as with data on higher respiratory metabolism in short‐lived genotypes of *C. elegans* (Feng et al., 2001; Lee et al., 2010).

The most significant expansion of lifespan occurred in *Daphnia* switched from the CMIH treatment to normoxia at the age of 30 days. Because this switch treatment included only one replicate tank, this result is highly speculative. In particular, the difference between switch and CMIH treatments in clones from intermittent habitats is suspect because it became apparent (Figure 1) even before the switch and thus represents random variation. It was also not significant in the switch vs. CMIH contrast for intermittent habitat clones, in contrast to all other contrasts. With this caveat in mind, if we were to believe that early life mild intermittent hypoxia provides a greater life‐extending effect than lifetime exposure, this may indicate that protective hypoxia‐induced pathways must be activated before aging‐related damages have started to accumulate or before the mid‐life transcriptional profile takes shape.

The late‐life increase in fecundity observed in CMIH treatment across all clones is intriguing. We have documented such late‐life fecundity spikes in several independent experiments (L. Yampolsky, C. Anderson, R. Lowman et al., in preparation), but the difference between CMIH and normoxia control observed here requires further confirmation, as it is based on a single day egg count and therefore may be spurious.

### 4.2 Chronic mild intermittent hypoxia does not increase acute hypoxia tolerance

Mild levels of stress are often supposed to acclimate or prepare organisms to tolerate higher, perhaps lethal levels of the same or related stress factor. However, the hormesis principle (Berry 3rd & López‐Martínez, 2020; Mattson, 2007; Maynard, 2011), should not be taken for granted (Axelrod et al., 2004). For example, in *Daphnia*, while adaptive effects of moderate heat acclimation have been well established (Burton et al., 2020; Yampolsky et al., 2014), the present study does not show a similar effect for moderate hypoxia. To the contrary, *Daphnia* reared in mild intermittent hypoxia showed lower survival in acute trials. Sometimes what does not kill us just makes us weaker. That is not to say that no acclimatory changes occur in hypoxia: beneficial effects of mild hypoxia in heat‐ and oxidative stress tolerance have been documented in *Daphnia* (Coggins et al., 2017; Klumpen et al., 2017). However, this plasticity appears to convey no benefit in terms of surviving in acute hypoxia.

### 4.3 Adaptive transcriptional responses to acute hypoxia

We observed several readily interpretable changes in acute hypoxia treatment that are likely to improve survival and to be based on a specific environment‐sensing mechanism. Apart from hemoglobin precursors and mitochondrial 5‐aminolevulinate synthase, a rate‐limiting enzyme in the heme synthesis pathway, transcripts that were upregulated in the ASH condition included two key enzymes in pyruvate metabolism: Ldh and PEPCK‐C. Ldh accomplishes anaerobic fermentation recycling NAD+, which allows ATP production to continue via glycolysis. PEPCK‐C is the rate‐limiting enzyme in early stages of gluconeogenesis and thus is critical for ATP production in gluconeogenesis‐dependent tissues such as muscles via the Cori cycle. Upregulation of UTP‐glucose‐1‐phosphate uridylyltransferase (UGP) is also consistent with these metabolic changes, assuming that in the shortage of glucose‐6‐phosphate and glucose‐1‐phosphate, it can replenish glucose‐1‐phosphate from glycogen storage. The upregulation of calcium‐binding mitochondrial carrier protein Aralar1 is also consistent with the apparent importance of gluconeogenesis, as its function is the transport of aspartate from the mitochondria to the cytosol, where it can be converted to oxaloacetate, the starting substrate for gluconeogenesis. Finally, the upregulation of sphingosine kinase is also consistent with the expected activation of HiF‐regulated pathways, because the product of sphingosine kinase, sphingosine 1‐phosphate, is known to be the key signal molecule in a pathway that interacts with HiF transcription factors and, in humans, determines myocyte survival during hypoxia and reperfusion (Karliner, 2009) and solid tumors' growth in hypoxic conditions (Cuvillier & Ader, 2011; Cuvillier et al., 2013).

Upregulation of gluconeogenesis by hypoxia is, on the first glance, paradoxal. The connection between hepatic hypoxia and gluconeogenesis has been recently documented in mammals (Ramakrishnan et al., 2016; Ramakrishnan & Shah, 2017) and fish (Sun et al., 2020), but the direction of change is exactly the opposite: hypoxia downregulates gluconeogenesis through three different pathways, all critically linked to HiF2α. Yet, in a recent studies in *C. elegans* (Vora et al., 2021), oysters (Le Moullac et al., 2007), and shrimps (Reyes‐Ramos et al., 2018; Xu et al., 2022) demonstrate, upregulation of PEPCK‐C also occurs in hypoxia, indicating that upregulation of gluconeogenesis in hypoxia may be a widespread phenomenon in invertebrates. What may be the functional significance of this reverse effect of hypoxia on gluconeogenesis? We believe that the key difference is in the heterogeneity of oxidation levels among tissue that exists in vertebrates. Notably, the liver is in a normoxic state during fasting and becomes hypoxic after refeeding when central blood flow is redirected toward the gastrointestinal tract (Ramakrishnan et al., 2016). This signals downregulation of the glucagon pathway and thus gluconeogenesis in the liver, contributing to the maintenance of blood glucose homeostasis. In animals such as *C. elegans* or *Daphnia*, this mechanism is not likely possible due to little or no lymph circulation regulation and a small body size, which probably results in a uniform oxygen availability throughout the body. In *Daphnia*, oxygen concentration in tissues shows an anterior–posterior gradient (less pronounced when hemoglobins are present in hemolymph) that reflects the general direction of circulation (Pirow et al., 2001). Thus, rather than switching between up‐ and downregulation of gluconeogenesis in response to feeding/fasting phases, hypoxia upregulates gluconeogenesis to resupply glucose for glycolysis in critically important tissues such as heart and striated muscles, to provide substrates for the pentose phosphate pathway, and to replenish NADPH that can be utilized in glutathione biosynthesis (Vora et al., 2021), thus contributing to the reduction of oxidative stress. This mechanism is fully corroborated by the shrimp studies (Reyes‐Ramos et al., 2018; Xu, Fu, et al., 2021) with gluconeogenesis‐related genes showing upregulation in hepatopancreas, but not in muscles. Elsewhere (Malek et al., in preparation), we report qPCR confirmation of this result with expression analyzed separately in heads and bodies of hypoxia‐exposed *Daphnia* (where, respectively, the majority of locomotory muscles, and the fat body, a likely location of gluconeogenesis, are located). These findings emphasize that the gluconeogenesis response to hypoxia is likely to be conserved among many animal lineages—as long as multicellularity allows compartmentalization necessary for the Cori cycle to operate.

It may be noted that transcripts upregulated specifically in clones from intermittent habitats (low number of clones per habitat type caveat notwithstanding) are dominated by catabolism enzymes encoding transcripts: ribonucleases, amylases, and peptidases, notably carboxypeptidases, with likely catabolic functions (Schwerin et al., 2009), indicating possible adaptive significance of activating ATP‐producing pathways alternative to aerobic respiration. This is consistent with previous findings of Zeis et al. (2009).

### 4.4 Transcriptional changes in response to hypoxia are not necessarily adaptive

Adaptive phenotypic plasticity (Ghalambor et al., 2007; Via et al., 1995) implies that a plastic response (1) reduces severity of negative or amplifies the effects of beneficial environmental factors and (2) is the result of past selection that created the mechanism of sensing environmental changes and specifically responding to them. By far not all cases of plasticity observed in nature fit this definition (Ghalambor et al., 2007). In this study there were numerous transcripts responding to acute hypoxia for which there was no such obvious adaptive explanation, or which, in fact, showed a stronger response in hypoxia‐sensitive than in hypoxia‐tolerant clones. There may be several reasons for this observation that can apply not just to our model organism. Firstly, some of these changes may be constrained, passive downstream responses to hypoxia (cf. Yampolsky et al., 2014) rather than adaptive ones. The nature of such responses may have to do with common regulatory pathways that are shared for entirely different reasons rather than adaptation to hypoxia, as seen, for example, in salt tolerance responses in yeast (MacGilvray et al., 2018). Alternatively, a transcriptional or phenotypic response to stress may simply occur because stress disrupts regulatory pathways in a constrained manner, as it is known, for example, for mitochondrial quality control pathways disrupted by oxidative stress (Ambekar et al., 2021). While these non‐adaptive responses can potentially be used as markers of hypoxic stress, they do not ameliorate damage caused by hypoxia. This is possibly the scenario we observe in the cases of some upregulated proteins with no obvious hypoxia‐ or respiration‐related functionality. The PCA analysis indicating that hypoxia‐tolerant clones become more transcriptionally similar to hypoxia‐sensitive ones when exposed to the ASH treatment and not vice versa, is suggestive that many transcriptional changes are indeed non‐adaptive.

Secondly, some of the transcriptional or phenotypic changes may be occurring in the adaptive direction but are not sufficient in magnitude relative to the strength of the stress factor. This is perhaps what we observe for the transcriptional responses to CMIH of globins or antioxidant enzymes encoding genes. Despite an arguably adaptive upregulation of these transcripts in the CMIH treatment, they, however, do not convey a higher tolerance to the ASH treatment (Figure 7a–d).

Finally, one may observe a phenotypic or transcriptional difference that is higher in sensitive individuals than in tolerant ones (Figure 7). This may reflect the higher internal damage experienced by the sensitive organisms rather than the higher adaptive value of the plastic response. We hypothesize that the stronger upregulation of egl‐9 homolog (HIF‐PH) by the ASH treatment in clones from permanent habitats than in clones from intermittent habitats reflects a greater degree of oxygen shortage in tissues (perhaps due to a lack of some other protective or compensatory mechanisms). Likewise, the upregulation of MAPK, the key protein in a general stress signaling pathway, that we observe in clones from permanent, but not intermittent habitats, may be explained by the same conjecture. A similar upregulation of MAPK has recently been observed in a relatively hypoxia‐sensitive carp species (Zhou et al., 2020). We have previously demonstrated similar, on the first glance counter‐intuitive, expression patterns in *Drosophila* in response to alcohol (Yampolsky et al., 2012) and in *Daphnia* in response to heat (Yampolsky et al., 2014).

Generally, these data emphasize the difficulties in observing preconditioning acclimatory effects and their transcriptional basis. The preconditioning stressor needs to be strong enough to cause a response, but not so strong as to be harmful, and the subsequent more severe stressor needs to not be so strong as to overwhelm the putative defenses provided by the acclimation.

### 4.5 Functional enrichment

Enrichment analyses based on a priori expected hypoxia‐related pathways and functions and on gene ontologies produced different results. Among a priori known hypoxia‐related pathways (Table 9), predictably, genes involved in antioxidant pathways, hemoglobins and cytoglobins, and HIF pathway‐related genes were enriched among transcripts responding to all treatments, while membrane phosphorylation‐related genes were enriched only among those responding to the ASH treatment. Other a priori known functionalities were enriched only in a subset of gene lists: for example, vitellogenins were only enriched among transcripts responding to ASH in clones from permanent habitats; HSPs and TNF pathway‐related genes—only among transcripts responding to CMIH in clones from intermittent habitats (Table 9). Despite their high occurrence in the reference, the autophagy‐related and mTOR‐related genes showed no enrichment in any of the analyses.

None of these a priori identified functional groups were identified in the GO enrichment analysis. In contrast, two other groups of genes appeared to be non‐randomly associated with hypoxia response: cuticular proteins and neurotrophins. In both these gene groups, the same pattern of ASH‐by‐habitat of origin interaction was observed: they were upregulated in the ASH treatment in the clones from intermittent habitats and downregulated in the clones from permanent ones (Figure 8). For cuticular proteins, one may hypothesize that hypoxia‐adapted *Daphnia* from the intermittent habitats respond to hypoxia by accelerating the molting cycle—a possible mechanism to increase the uptake of oxygen in hypoxic conditions due to the higher oxygen penetrability of soft post‐molt exoskeleton (Mangum et al., 1985; Peruzza et al., 2018). Peruzza et al. (2018) also observed changes in cuticular protein expression in response to hypoxia. On the other hand, it is less obvious why hypoxia‐sensitive clones downregulate cuticular proteins; perhaps it is a part of a general trend to conserve resources by downregulating nonessential gene expression.

Much less can be concluded from the same patterns observed in neurotrophins. First of all, this enrichment may be spurious as these genes are closely linked and may be under a common transcriptional regulation, thus representing a single independent transcriptional response, rather than six. However, if indeed not caused by chance alone, the radically different responses to acute hypoxia observed in hypoxia‐tolerant and hypoxia‐sensitive clones suggest an intriguing (albeit highly speculative) possibility that survival in hypoxia might depend on preventing neuronal death. Neurotrophins have known functions in maintaining neuron survival (Hempstead, 2006). Possible role of neurotrophic factors and stress‐response pathways in preventing neurons damage during hypoxia has been previously discussed (Bickler, 2004), and recent data indicate that their well‐characterized transmembrane p75 receptor plays a crucial role in neuronal death and survival during oxidative stress and hypoxia (Sankorrakul et al., 2021). A detailed characterization of the hypoxia response of *Daphnia* neurotrophin and neurotrophin receptor genes is needed to investigate this potentially interesting tolerance mechanism.

## 5 CONCLUSIONS

Chronic mild intermittent hypoxia (CMIH) extended lifespan in hypoxia‐tolerant, high mitochondrial membrane potential clones from intermittent habitats that are characterized by a shorter lifespan in normoxic conditions. It slightly shortened the lifespan of permanent habitat clones with longer normoxic lifespan. The CMIH treatment did not precondition *Daphnia* to better survive acute severe hypoxia (ASH) and resulted in few detectable transcriptional changes. Transcriptional changes associated with higher ASH survival include genes in pathways related to oxygen transport and storage, lactate and pyruvate metabolism, gluconeogenesis, and, specifically in hypoxia‐tolerant clones, catabolism pathways, cuticulum proteins, and neurotrophins, indicating roles of anaerobic respiration, gluconeogenesis, modulating molting cycle, and maintaining neuronal survival in hypoxia tolerance.

## AUTHOR CONTRIBUTIONS

**Millicent N. Ekwudo:** Conceptualization (equal); investigation (equal); project administration (equal); writing – review and editing (equal). **Morad C. Malek:** Investigation (equal); validation (equal). **Cora E. Anderson:** Investigation (equal); validation (equal). **Lev Y. Yampolsky:** Conceptualization (equal); data curation (equal); formal analysis (equal); funding acquisition (lead); investigation (equal); methodology (equal); project administration (equal); resources (equal); supervision (lead); visualization (equal); writing – original draft (equal).

## CONFLICTS OF INTEREST

Authors declare no conflicts of interest.

## Supporting information


Figures S1–S4

## Data Availability

RNA reads and differential expression data are available from NCBI, GSE207385. Quantitative data necessary for recreating all analyses reported will be archived in DataDryad depository (https://doi.org/10.5061/dryad.p5hqbzkrt).
