# Supplementary material for: The interplay between prior selection, mild intermittent exposure, and acute severe exposure in phenotypic and transcriptional response to hypoxia
Source: Ecol Evol. 2022 Oct 9;12(10):e9319. doi: 10.1002/ece3.9319 (PMC9548574; doi:10.1002/ece3.9319)
Supplement: Supplementary file 1 — Figures S1–S4 [file ECE3-12-e9319-s001.docx]

Supplementary Figures


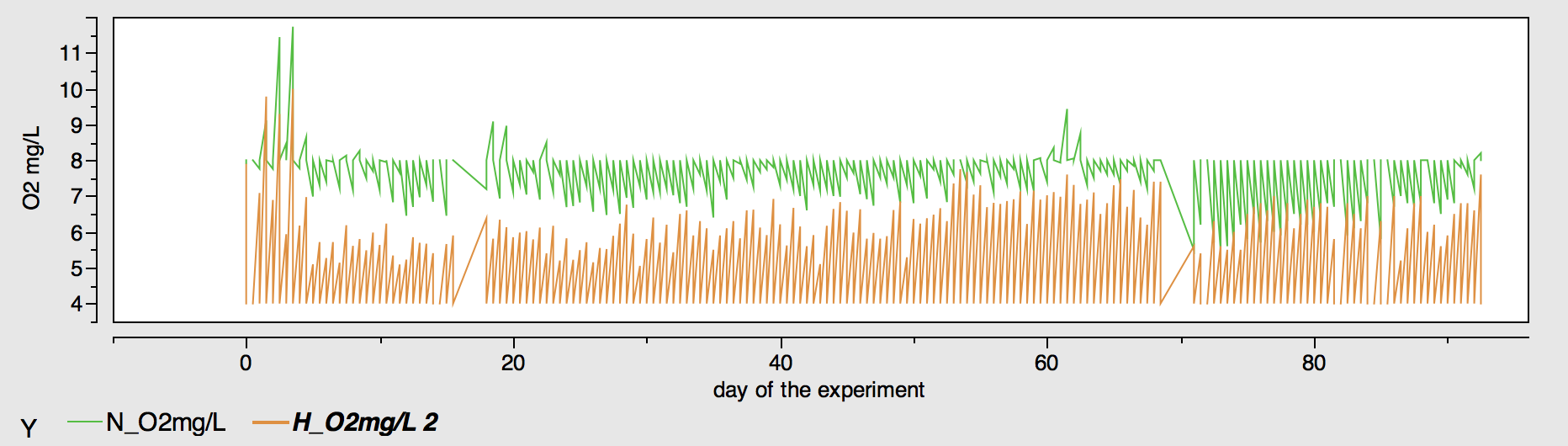


Fig. S1. DO concentrations in the CMIH experiment (a random tank selected for the illustration purposes) showing typical span of daily DO in normoxic treatment (green) and chronic mild intermittent hypoxia treatment (orange). The graph shows three episodes of oversaturation and two brief periods during which DO measurements were not recorded (although DO adjustments were done).

**Summary of Fit**


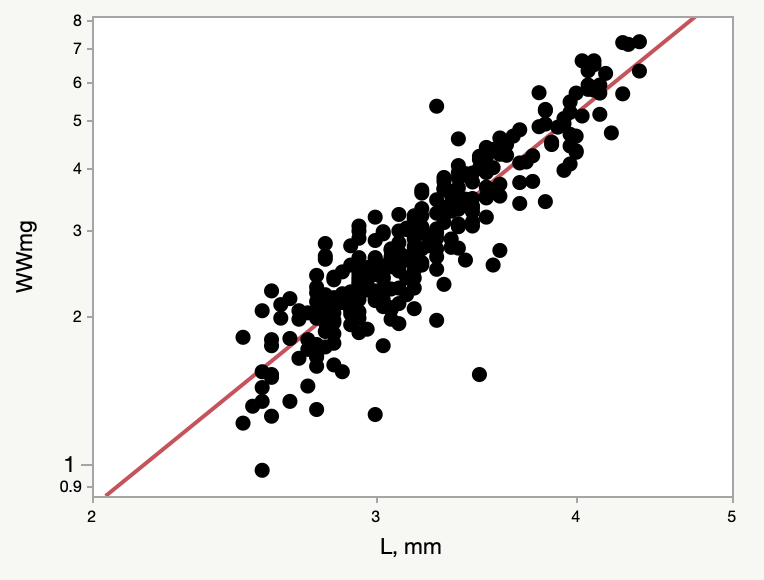


Log(WW,mg) = -2.05 + 2.66*Log(L,mm)

| RSquare | 0.818993 |
| --- | --- |
| RSquare Adj | 0.818409 |
| Root Mean Square Error | 0.157644 |
| Mean of Response | 1.037977 |
| Observations (or Sum Wgts) | 312 |

**Parameter Estimates**

| **Term** | **Estimate** | **Std Error** | **t Ratio** | **Prob>\|t\|** |
| --- | --- | --- | --- | --- |
| Intercept | -2.0488 | 0.0829 | -24.71 | <.0001* |
| Log(L, mm) | 2.6630 | 0.0711 | 37.45 | <.0001* |

Fig. S2. Wet weight to body length power law regression used to estimate body weight for respiration and feeding rate normalization purposes. Data from Coggins et al. 2017. Same clones of *D.magna* grown in similar conditions were used to obtain the regression.

A B

Read length log10(bp)

Read length log10(bp)

C

Fig S3. Distribution of read lengths in a typical sequencing run (A,B)and total numbers of reads analyzed per sample (C). A: passed reads; B: failed reads. Failed reads enriched in very short reads <150 bp and very long reads (over 10kbp). Box-and-whiskers plots show quantiles, mean, quartiles +/- 1.5 interquartile range whiskers, the shortest half bracket (red, is range containing the most dense 50% of observations), and outliers. C: N, CMIH normoxia treatment (green); H, CMIH hypoxia treatment (orange); C, ASH control (no outline); A, ASH acute hypoxia treatment (outline).

Fig. S4. Lifespan of *Daphnia* from intermittent habitats (dotted lines) and permanent habitats (solid lines) in normoxic conditions (A; green), chronic mild intermittent hypoxia (4 mg O_2_ /L twice daily; B; orange), or after a switch from 4 to 8 mg O_2_ /L at day 30. P values for Log-rank test for survival differences between groups are shown. See Table 1 for detailed survival analysis. See main text Fig.1 for the same data grouped by habitats of origin rather than hypoxia conditions.

P<0.0001

P>0.17

P>0.44

A

B

C
